# Ocular and inflammatory markers associated with Gulf War illness symptoms

**DOI:** 10.1038/s41598-023-30544-9

**Published:** 2023-03-02

**Authors:** Karthik Kalahasty, Yonghoon Lee, Elyana Locatelli, Mak Djulbegovic, Kimberly Cabrera, Parastou Pakravan, Courtney Goodman, Andrew Jensen, Kristina Aenlle, Nancy Klimas, Raquel Goldhardt, Anat Galor

**Affiliations:** 1 University of Miami Miller School of Medicine, Miami, FL USA; 2 Ophthalmology, Miami Veterans Affairs Medical Center, 1201 NW 16th St, Miami, FL 33125 USA; 3 Nova Southeastern University, Ft Lauderdale, FL USA; 4 Bascom Palmer Eye Institute, University of Miami, Miami, FL USA; 5 Research Services, Miami Veterans Affairs Medical Center, Miami, FL USA

**Keywords:** Biomarkers, Biomarkers, Medical research

## Abstract

To examine the utility of ocular coherence tomography (OCT) metrics, in conjunction with systemic markers of inflammation, in identifying individuals with Gulf War Illness (GWI) symptoms. Prospective case–control study of 108 Gulf War Era veterans, split into 2 groups based on the presence of GWI symptoms, defined by the Kansas criteria. Information on demographics, deployment history, and co-morbidities were captured. 101 individuals underwent OCT imaging and 105 individuals provided a blood sample which was analyzed for inflammatory cytokines using an enzyme-linked immunosorbent assay-based chemiluminescent assay. The main outcome measure was predictors of GWI symptoms, examined with multivariable forward stepwise logistic regression analysis followed by receiver operating characteristic (ROC) analysis. The mean age of the population was 55 ± 4, 90.7% self-identified as male, 53.3% as White, and 54.3% as Hispanic. A multivariable model that considered demographics and co-morbidities found that a lower inferior temporal ganglion cell layer-inner plexiform layer (GCL‒IPL) thickness, higher temporal nerve fiber layer (NFL) thickness, lower interleukin (IL)-1β levels, higher IL-1α levels, and lower tumor necrosis factor-receptor I levels correlated with GWI symptoms. ROC analysis demonstrated an area under the curve of 0.78 with the best cut-off value for the prediction model having a sensitivity of 83% and specificity of 58%. RNFL and GCL‒IPL measures, namely increased temporal thickness and decreased inferior temporal thickness, respectively, in conjunction with a number of inflammatory cytokines, had a reasonable sensitivity for the diagnosis of GWI symptoms in our population.

## Introduction

Years after the Gulf War (1990–1991), many veterans reported a wide range of symptoms including fatigue, mood disorders, cognitive disorders, integumentary manifestations, gastrointestinal disturbances, and musculoskeletal disorders^1,2^. This constellation of symptoms has since been classified as Gulf War Illness (GWI). Exposure to chemicals and adverse environmental conditions during the Gulf War (i.e., sarin, pesticide sprays, pyridostigmine bromide (PB) prophylactic pills, tent heater exhaust) triggering neuroinflammation and subsequent neuronal abnormalities is hypothesized to be one contributor to GWI symptoms^2,3^. However, exposures in the Gulf arena cannot be the sole contributor to GWI symptoms as some veterans who were active but not deployed during the Gulf War Era (GWE) report a similar constellation of symptoms. The systemic findings and lack of clear etiology associated with GWI symptoms demonstrate that the disease is complex and requires further investigation. A better understanding of disease pathophysiology can facilitate clinical diagnosis and uncover therapeutic targets.

Neuroinflammation as a basis of GWI symptom development has been suggested by numerous studies. For example, one study examined corticomotor excitability in 40 deployed GWE veterans, grouped by presence of GWI related chronic pain. Veterans with GWI related diffuse pain (n = 20) had a higher average resting motor threshold compared to controls (n = 20) (77.2 ± 16.7% vs. 55.6 ± 8.8%, *p* < 0.001), implying diminished corticomotor excitability in veterans with GWI related diffuse pain^4^.

The specific intermediaries of chronic neuroinflammation in GWI remain under investigation, with variable findings across studies. For example, in one study, veterans with GWI (n = 25) had serum elevations of interleukin 4 (IL-4) and IL-13 compared to 28 GWE veteran controls without GWI (53% of GWI cases had levels > 20% higher than controls)^5^. However, another study that measured levels of 77 serum cytokines found lower levels of IL-4 (0.9 vs. 1.1 pg/mL, *p* = 0.04) and IL-13 (8.2 vs. 9 pg/mL, *p* = 0.001) in 37 veterans with GWI compared to 42 civilian controls^6^. As such, further characterization of inflammatory markers within the context of imaging studies is required in GWI.

Functional magnetic resonance imaging (fMRI) is one technique applied to the study of GWI. In one study of 96 individuals with GWI and 44 controls, cases had markedly decreased pre-frontal cortex activity with memory tasks compared to controls^7^. This study provided a neurological correlate to the cognitive deficits reported by individuals with GWI. Structural MRI scans have also provided a correlate for central neurodegeneration in GWI. One study of 111 veterans with GWI and 59 controls demonstrated significant subcortical atrophy in the case group, primarily in brainstem volume^8^. Another study of 90 veterans with GWI and 34 controls noted reduced gray matter volumes in the frontal, parietal, and occipital lobes in the case group^9^. However, limitations of MRI and fMRI include time, expense, and expertise of scanning and interpreting images. As such, other technologies are needed to examine neural status in GWI.

Optical coherence tomography (OCT) is one such possible technology as it is a non-contact imaging technique that is readily available in all eye clinics. OCT utilizes infrared light to produce high-resolution, cross-sectional images of the retina and optic nerve with built-in software that quantifies the retinal nerve fiber layer (RNFL) and ganglion cell layer-inner plexiform layer (GCL‒IPL) thickness^10–12^. Prior studies have examined RNFL and GCL‒IPL thicknesses in a variety of neurodegenerative and neuroinflammatory disorders, including Alzheimer’s disease (AD), Parkinson’s disease (PD), and Multiple Sclerosis (MS)^1,13,14^. For example, a study in 119 patients with MS (65 with optic neuritis, 52 without optic neuritis) found that RNFL and GCL‒IPL thinning observed on OCT were significantly correlated with visual acuity in a low contrast environment (RNFL: R^2^ = 0.31, *p* = 0.001, GCL‒IPL: 0.28, *p* = 0.03)^15^. Interestingly, OCT measures have also been correlated with brain-substructure and grey and white matter volumes in MS. These findings suggest that OCT may non-invasively monitor brain status^16^. Ocular imaging, therefore, provides a tool to easily image central nerves that may be applied to the study of GWI. In this study, we examine the utility of OCT measures, in conjunction with plasma inflammatory markers, to identify individuals with GWI symptoms. These data have the potential to aid in disease diagnosis and monitoring.

## Methods

### Study population

We performed a prospective study of 108 veterans who served during the GWE and who were seen in an eye clinic at the Miami Veterans Affairs (VA) Hospital between November 2018 and February 2022. Inclusion criteria included veterans who were active during 1990–91, both deployed and non-deployed. Exclusion criteria included active infection or pregnancy, current psychotic disorder not managed for more than 6 months with a stable treatment regimen, history of head injury with loss of consciousness greater than 5 min, significant neurological disorders outside of the scope of GWI, or any medical conditions that would make study procedures difficult. Furthermore, individuals with eye pathology that may impact OCT imaging (e.g., diabetic retinopathy, glaucoma) were excluded from participation. Informed consent was obtained from all the patients that participated in the study. The study was approved by the Miami VA Institutional Review Board (IRB). The study was conducted in accordance with the principles of the Declaration of Helsinki and complied with the requirements of the United States Health Insurance Portability and Accountability Act.

### Capture of GWI symptoms

Our analysis focused on individuals with GWI symptoms as diagnosed with the Kansas questionnaire survey (Supplemental Fig. 1). Individuals were considered as having GWI symptoms if they had one severe or two moderate symptoms in at least three of six domains, including (1) fatigue, (2) pain, (3) neurologic and mood, (4) gastrointestinal, (5) respiratory, and (6) skin^17^. Veterans were divided into 2 groups based on GWI symptoms status (yes/no). Individuals were further grouped based on deployment status (yes/no). Of the 108 individuals included in the study, 37 met the Kansas criteria for GWI symptoms and 71 served as age-matched controls.

### Data collection

Data on demographics, co-morbidities, medications, and medical and ocular diagnoses were collected from all individuals.

### Imaging

101 individuals underwent OCT imaging using a Cirrus HD-OCT (Carl Zeiss Meditec Inc, Dublin, California, USA). The specific OCT parameters captured were the retinal nerve fiber layer (RNFL), GCL‒IPL, and macular maps. To account for correlation between eyes, new variables representing the (a) thicker and (b) thinner values for either eye were calculated and included in the statistical models.

### Laboratory data

105 individuals provided a blood sample (K2EDTA blood tubes) on the day of imaging from which cytokines levels were measured in plasma. In short, samples were processed within two hours of the blood draw and plasma was collected by centrifugation at 2000 × g for 15 min and stored at − 80 °C. Cytokine analysis was performed using a custom 18-multiplex chemiluminescent assay (Quansys). The cytokine panel included Tumor Necrosis Factor alpha (TNFα), TNFβ, IL-1α, IL-1β, IL-2, IL-5, IL-6, IL-8, IL-10, IL-12, IL-13, IL-15, IL-17, IL-23, TNF-RI, TNF-RII, and IFN-g. Plasma samples were thawed at 4 °C overnight and plated in duplicate following the manufacture's protocol. The 96-well plates were read at a range of exposure times using the Q-view Imager LS (Quansys). Individual cytokine concentrations were obtained using image analysis software (Q-View v3.13, Quansys Bioscience). Sample concentrations were calculated from standard curves created using best fit logistic regression. Values of cytokine concentration levels are reported in pg/ml. Internal controls were used to normalize between plates.

### Data analysis

Statistical analyses were performed using SPSS 28.0 (IBM Corp, Armonk, NY). We used descriptive statistics to summarize demographic and clinical data. Student’s t-test and analysis of variance (ANOVA) were used to compare mean differences in continuous variables between groups (n = 108). Predictors of GWI-related symptoms were analyzed using forward stepwise binary logistic regression and receiver operating characteristic (ROC) curves for individuals that had both cytokine data and OCT imaging data (n = 99). The binary logistic regression analysis was repeated for deployed versus non-deployed individuals and finally for deployed individuals with GWI symptoms versus deployed controls.

### Conference presentation

Meeting Presentations: ARVO Annual Meeting—May 2022, Denver, CO; AAO Annual Meeting—September–October 2022, Chicago, IL

## Results

### Study population

The mean age of the population (n = 108) was 55 ± 4, 90.7% self-identified as male, 53.3% as White, and 54.3% as Hispanic. Demographics were similar between those with vs. without GWI symptoms. Individuals with GWI symptoms were more likely to be diagnosed with hyperlipidemia (74% vs. 46%, *p* = 0.007) and post-traumatic stress disorder (PTSD, 49% vs. 29%, *p* = 0.06), and to use an anti-depressant (43% vs. 24%, *p* = 0.06) (Table 1). Given that individuals with GWI symptoms had a higher incidence of hyperlipidemia, BMI was controlled for in the subsequent multivariable analysis. Furthermore, diagnosis of PTSD and anti-depressant use was controlled for in the multivariable analysis (Table 2).

**Table 1:** Demographics and clinical data grouped by presence of Gulf War Illness (GWI) symptoms.

	GWI symptoms (n = 37)	No GWI symptoms (n = 71)	*p*-value
Demographics			
Age, mean ± SD	55 ± 4.7 years	56 ± 5.0 years	0.86
Sex, male % (n)	95% (35)	87% (62)	0.49
Race, White % (n)	64% (24)	49% (35)	0.44
Black % (n)	36% (13)	43% (31)	
Ethnicity, Hispanic % (n)	42% (16)	47% (34)	0.55
Deployed, Yes % (n)	81% (30)	52% (37)	0.003
BMI, mean ± SD	28.97 ± 4.63 kg/m^2^	30.81 ± 6.43 kg/m^2^	0.23
Co-morbidities, n (%)			
Hyperlipidemia*	74% (27)	46% (33)	0.007
Sleep Apnea	71% (26)	54% (38)	0.10
PTSD	49% (18)	29% (21)	0.06
Depression	46% (17)	39% (28)	0.52
Hypertension	46% (17)	41% (29)	0.68
Arthritis	40% (15)	41% (29)	0.90
Hepatitis C	2.9% (1)	1.7% (1)	0.70
Benign prostatic Hyperplasia	21% (8)	17% (12)	0.69
Medication use, n (%)			
Anti-depressant	43% (16)	24% (17)	0.06
Statin	40% (15)	45% (32)	0.65
Multivitamin	37% (14)	34% (24)	0.75
NSAID	34% (13)	38% (27)	0.72
Antihistamine	26% (10)	31% (22)	0.58
Beta blocker	20% (7)	16% (11)	0.58
Aspirin	17% (6)	21% (15)	0.68
Anti-anxiety	14% (5)	16% (9)	0.87
Gabapentin	11% (4)	14% (10)	0.74
Sildenafil	9% (3)	21% (15)	0.12

*SD* Standard deviation, *n* Number in group, *PTSD* Post-traumatic stress disorder, *NSAID* Nonsteroidal anti-inflammatory drug, *BMI* Body mass index.

**Table 2:** Multivariable logistic regression model examining factors predictive of Gulf War Illness (GWI) symptoms.

Variable	OR	95% CI	*p*-value
Temporal NFL thickness	1.07	1.01–1.12	0.01
Inferior temporal GCL‒IPL thickness	0.88	0.81–0.96	0.003
Interleukin-1β	0.78	0.66–0.93	0.004
Interleukin-1α	1.21	1.06–1.39	0.006
Tumor necrosis factor receptor I	0.995	0.991–0.999	0.02

*OR* Odds ratio; *CI* Confidence Interval; *NFL* Nerve fiber layer; *GCL*‒*IPL* Ganglion cell layer-inner plexiform layer.

### OCT and inflammatory markers associated with GWI symptoms

Individuals with (n = 37) vs. without GWI symptoms (n = 71) had higher temporal NFL thickness when examining both the thicker value (68.44 ± 22.76 vs. 61.14 ± 10.02 µm, *p* = 0.07) or thinner value (61.33 ± 11.52 vs. 55.80 ± 10.23 µm, *p* = 0.02) of either eye. Additionally, individuals with GWI symptoms had lower IL-1β levels (11.1 ± 4.1 vs. 13.6 ± 5.8 pg/mL, *p* = 0.02) and lower TNF-RI levels (418.1 ± 157.6 vs. 481.3 ± 173.2 pg/mL, *p* = 0.07) compared to controls.

### Relationships between OCT measures, inflammatory markers, and GWI symptoms

**Figure 1:**
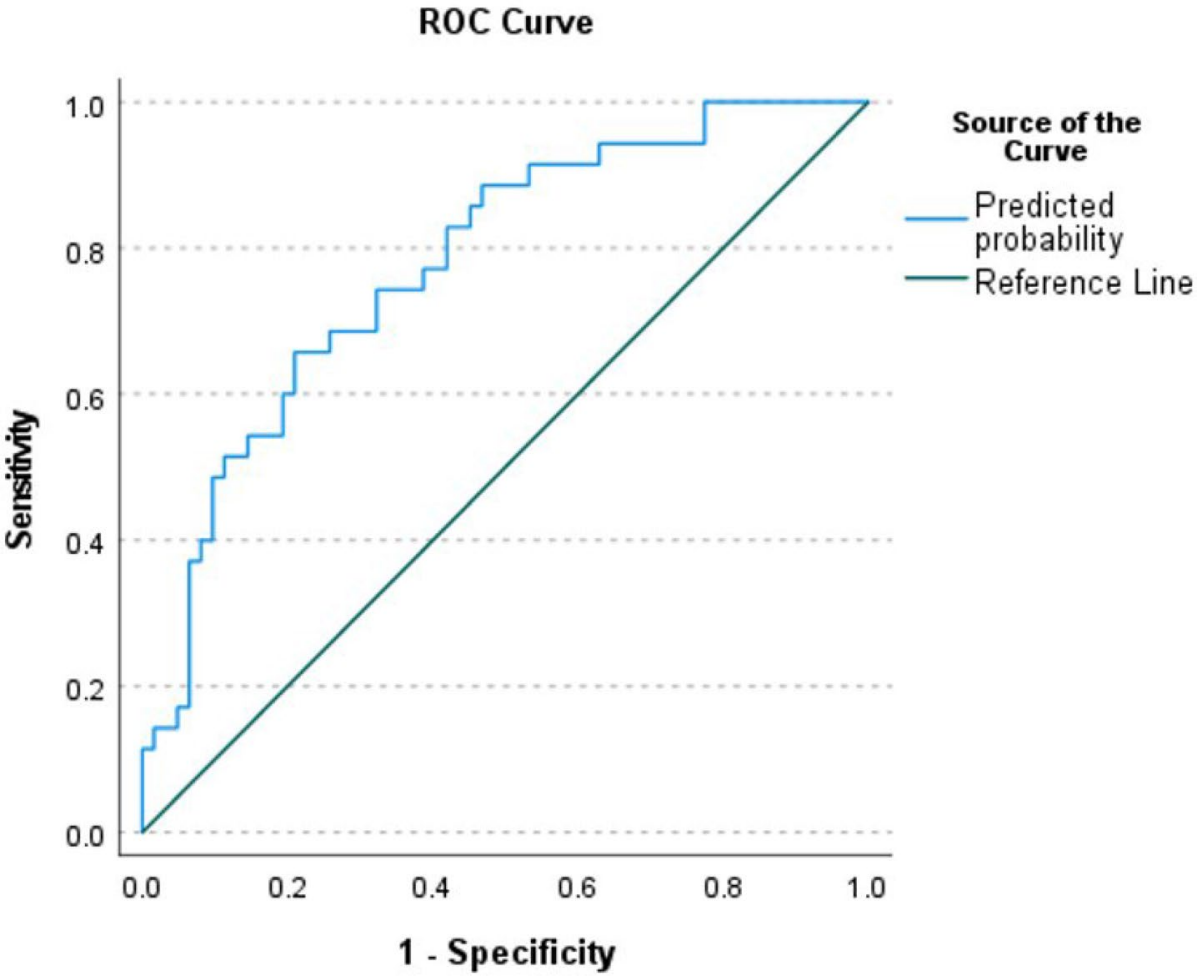
Receiver operating characteristic curve of model predictive of Gulf War illness symptoms which includes both optical coherence tomography measurements and inflammatory markers.

After confirming non-collinearity between predictors, a stepwise multivariable logistic regression analysis model (n = 99), which included demographics and co-morbidities, found that lower inferior temporal GCL‒IPL thickness (odds ratio; OR = 0.88, 95% confidence interval; CI = 0.81–0.96, *p* = 0.003), higher temporal NFL thickness (OR = 1.07, 95% CI = 1.01–1.12, *p* = 0.003), lower IL-1β levels (OR = 0.78, 95% CI = 0.66–0.93, *p* = 0.004), higher IL-1α levels (OR = 1.21, 95% CI = 1.06–1.39, *p* = 0.006), and lower TNF-RI levels (OR = 0.995, 95% CI = 0.991–0.999, *p* = 0.015) remained predictive of GWI symptoms (Table 2). ROC analysis demonstrated an area under the curve of 0.78 (95% CI = 0.69–0.87, *p* < 0.001) for this model (Fig. 1). The best cut-off value for the prediction model had a sensitivity of 83% and specificity of 58%, as determined by Youden’s index.

### Relationships between OCT measures, inflammatory markers, and deployment status

We next repeated the analysis to examine which variables identified in the primary model were also associated with deployment status (deployed vs. non-deployed, n = 99) (Table 3). Similar to the GWI symptom model (Table 2), lower IL-1β levels (OR = 0.93, 95% CI = 0.85–1.003, *p* = 0.06) remained predictive of deployment. Missing from this model were OCT measures, IL-1α and TNF-RI, suggesting that these biomarkers were more specific for GWI symptoms and not deployment status.

**Table 3:** Multivariable logistic regression model examining factors predictive of deployment and Gulf War Illness (GWI) symptoms in deployed veterans.

Comparison	Variable	Odds ratio	95% CI	*p*-value
Deployed (yes vs. no)	Interleukin-1β^a^	0.93	0.85–1.003	0.06
GWI symptoms (yes vs. no) in deployed veterans	Temporal NFL Thickness^a^	1.08	1.01–1.16	0.03
	Inferior GCL‒IPL Thickness	0.78	0.64–0.94	0.009
	Superior GCL‒IPL thickness	1.20	0.998–1.44	0.05

^a^Indicates variable that remained from primary analysis (GWI symptoms, yes vs. no, in entire population).

^b^*NFL* Nerve fiber layer, *GCL*‒*IPL* Ganglion cell layer-inner plexiform layer, *GWI* Gulf War Illness.

### Relationships between OCT measures, inflammatory markers, and GWI symptoms in deployed veterans

We next repeated the analysis (n = 64) to examine which factors identified in the primary model remained predictive of GWI symptoms within those deployed to the Gulf War arena (Table 3). Similar to our original model for GWI symptoms, a higher temporal NFL thickness remained predictive of GWI symptoms in deployed veterans compared to deployed controls (OR = 1.08, 95% CI = 1.01–1.16, *p* = 0.03). New variables in this model predictive of GWI symptoms were a decreased inferior GCL‒IPL thickness (OR = 0.78, 95% CI = 0.64–0.94, *p* = 0.009), and increased superior GCL‒IPL thickness (OR = 1.20, 95% CI = 0.99–1.44, *p* = 0.05). The OCT measure in the original model and not in the deployed model was decreased inferior temporal GCL‒IPL thickness. There were no inflammatory markers that were predictive of GWI symptoms when examining only deployed veterans. ROC analysis demonstrated an area under the curve of 0.64 (95% CI = 0.51–0.76, *p* < 0.001) for this model. The best cut-off value for the prediction model had a sensitivity of 66% and specificity of 54%, as determined by Youden’s index.

## Discussion

To conclude, we found that a combination of OCT measures and plasma inflammatory cytokine markers predicted GWI symptoms with reasonable sensitivity (83%) but low specificity (58%). The strongest signal was for higher temporal NFL and lower inferior temporal GCL‒IPL thicknesses which held both when examining the population as a whole and when only focusing on deployed veterans. Interestingly, lower interleukin-1β levels, which was the cytokine most strongly linked to GWI symptoms, seem to be more indicative of deployment status more so than symptoms. Furthermore, it is notable that the model which focused on GWI symptoms within deployed veterans was not as robust as the one which included all veterans (deployed and non-deployed). This highlights the need for further research on measures that more closely relate to GWI symptoms in deployed veterans.

Our current findings are different to that of our previous retrospective study of 28 individuals with GWI and 38 controls where we found overall RNFL thickness to be lower in individuals with a GWI diagnosis (OR = 0.95, *p* = 0.04)^1^. However, our groupings in the previous study were not identical to our current study. In the former study, we compared deployed veterans with GWI symptoms to controls which included both deployed veterans without GWI symptoms and all non-deployed GWE veterans. In the current study, after recognizing that non-deployed veterans can have a positive Kansas screen, we grouped individuals by presence of GWI symptoms, irrespective of deployment, and considered deployment status in subsequent models.

Our results share both similarities and differences with other neurodegenerative and neuroinflammatory disorders such as MS, AD, PD, and Huntington’s disease (HD)^18,19^. Unlike our findings of temporal RNFL thickening in GWI cases compared to controls, diffuse RNFL thinning has been noted in individuals with MS, both those with and without a history of optic neuritis, with the temporal quadrant most severely affected^18^. In a study of 546 MS cases and 157 controls, temporal RNFL thickness was significantly reduced in cases versus controls (− 9.35 μm mean difference between groups, *p* < 0.001), and was a more sensitive marker for predicting presence of MS compared to total average RNFL thickness (−7.58 μm mean difference between groups)^19^.

In fact, temporal RNFL thinning has been a consistent theme in OCT studies of neurodegenerative diseases. For example, a study of 26 individuals with HD found lower temporal RNFL thickness in those with versus without HD (62.3 vs. 69.8 μm, *p* = 0.005) and found a negative correlation between temporal RNFL thickness and disease duration (R^2^ =  − 0.51, *p* = 0.04)^20^. Similar findings have been described in PD. In a study of 43 individuals with PD and 86 controls, significant temporal RNFL thinning was noted in cases versus controls, with the eye contralateral to the more affected body side having thinner RNFL measurements than the contra-lateral eye^21^. This finding has also been observed in diseases with localized neuro-degeneration, such as Leber’s Hereditary Optic Neuropathy (LHON). In a study of 221 eyes of individuals with LHON compared with 52 control eyes, a distinct pattern of thinning was noted that first affected the temporal RNFL, followed by inferior, superior, and lastly nasal quadrant^22^. Comparatively, temporal RNFL thickness was increased in GWI cases versus controls with a magnitude of effect similar to that found in HD.

A potential explanation to the discrepancies noted above is the lack of longitudinal information in our and other populations. In fact, in LHON, initial RNFL thickening was noted in the acute phase (within 3 months of disease onset), followed by progressive RNFL thinning that appeared between 3 and 6 months after disease onset^22^. Similar findings have been described in MS with optic neuritis, where earlier in the disease, optic disc swelling due to optic neuritis may present with RNFL thickening^23^. RNFL thickening may be due to a number of factors including impaired axoplasmic transport with subsequent fluid stasis, overproduction of reactive oxygen species with induction of inflammatory edema, and microvascular changes that compensate for energy deficiency in the temporal sector^22,24^. As such, our population’s OCT findings may represent an earlier stage of disease, with thinning expected over time. Longitudinal studies are needed to test this hypothesis.

Interestingly, while temporal RNFL thinning is a late marker of disease in MS and AD, GCL‒IPL abnormalities have been described earlier in the disease course. For example, one study examined OCT parameters longitudinally in 23 individuals with MS and no history of optic neuritis and 10 controls^25^. At baseline, mean GCL‒IPL thickness was lower in the MS versus control group (75 vs. 82 μm, *p* = 0.008) but temporal RNFL thickness was similar between the groups. This indicates that GCL‒IPL thinning may be more sensitive disease marker in early stages of MS. Early GCL‒IPL abnormalities have also been observed in AD. In a study of 100 individuals with AD and 123 cognitively normal controls, GCL‒IPL thickness was diffusely thin in all 6 sectors (differences between groups ranged from −3.42 to −4.99 μm, *p* < 0.05)^26^. Similar findings were seen in PD. In a study of 54 individuals with PD and 54 controls, diffuse GCL‒IPL thinning was observed compared to controls (mean thickness 68.6 vs. 81.3 μm, *p* < 0.001)^27^. Our finding that inferior temporal GCL thinning was associated with GWI symptoms, albeit with a smaller difference in magnitude compared to MS, AD, and PD (Supplementary Table 1), is consistent with findings from other neurodegenerative diseases.

Several hypotheses have been proposed for the temporal location of involvement in neuro-degenerative diseases. This includes a higher ratio of small parvocellular axons to magnocellular axons in the temporal quadrant which are more susceptible to damage in addition to inefficient energy use by temporal RNFL and GCL‒IPL parvocellular axons that possess a small volume-to-surface area^19,28,29^. The hypothesis of inefficient energy use by these parvocellular axons is supported by temporal RNFL thinning in localized neurodegenerative disorders where mitochondrial function is compromised such as LHON^22,30^. Furthermore, the density of ganglion cells is lowest in the inferior and temporal quadrants rendering this region sensitive to neuro-degenerative changes^31^. Therefore, the association of temporal RNFL and GCL‒IPL differences in individuals with GWI symptoms versus controls may share a similar etiology.

Exposures in the Gulf arena may be a contributing factor to the temporal RNFL and GCL findings in our population. In a study of 80 GWE veterans, individuals exposed to sarin and cyclosarin (n = 40) had less total gray matter and hippocampal volumes on T1-weighted MRI compared to unexposed controls (n = 40)^32^. Decreased total gray matter in this study was correlated with diminished visuospatial function (Block Design test, r = 0.42, *p* = 0.01). Interestingly, parvocellular pathways are known to contribute to high spatial acuity, particularly in the thalamus^33^. This common parvocellular pathway provides a link between neurodegenerative changes observed on MRI in the thalamus and our noted OCT findings. Animal studies have further linked toxic exposures to abnormal function and neural anatomy. In one study, rats were treated with organophosphate chemicals for 4 weeks and assessed 5- and 10-months post-exposure. Exposed rats had impairments in multiple neurobehavioral tests, including memory deficits and aggressive behavior. Furthermore, magnetic resonance imaging (MRI) demonstrated decreased hippocampal and thalamic volume in exposed vs. non-exposed rats, linking neurodegeneration to the observed neurocognitive and neurobehavioral deficits^34^. These studies demonstrate that exposure to various toxins can lead to CNS abnormalities, which may later manifest with OCT findings.

Our data did not find strong relationships between inflammatory cytokines and GWI symptoms, with differences between cases and controls changing when deployment status was considered (Table 3). In fact, relationships between systemic inflammatory markers and GWI have varied across prior studies (Table 4). For example, a study of 15 veterans with GWI and 33 controls (8 deployed GWE veterans and 25 civilians) found no differences in plasma inflammatory cytokines (TNF-α, IL-6 and IL-1β) between groups^35^. On the other hand, a study of 37 veterans with GWI and 42 civilian controls found decreases in TNF-α (19.3 vs. 23.1 pg/mL, *p* < 0.001), IL-13 (8.2 vs. 9.1 pg/mL, *p* = 0.001), and IL-25 (1.3 vs. pg/mL 2.4, *p* = 0.04) and increases in IFN-γ (22.4 vs. 15.4 pg/mL, *p* = 0.003), IL-5 (6.4 vs. 5.9 pg/mL, *p* = 0.01), and IL-17F (47.8 vs. 17.9 pg/mL, *p* = 0.001) in cases versus controls^6^. Yet another case–control study of deployed veterans with (n = 8) and without (n = 5) GWI reported higher serum TNF-RI in cases vs controls (852.7 vs. 452.6 pg/mL, *p* = 0.04)^36^. By contrast, we found lower plasma TNF-RI in GWI cases versus controls, with a smaller difference in magnitude (418.1 ± 157.6 vs 481.3 ± 173.2 pg/mL, *p* = 0.07) and no differences when considering only deployed veterans. IL-1α has also been examined as an inflammatory marker of interest in GWI. Specifically, IL-1α levels were significantly higher at rest in one study attempting to characterize immune signaling change during exercise in 9 individuals with GWI and 11 healthy controls. In this study, the difference between plasma IL-1α levels in individuals with versus without GWI was marked prior to exercise (22.01 vs. 2.12 pg/mL, *p* < 0.001), with the association pattern disrupted during exercise^37^. Our study also found higher plasma IL-1α levels in individuals with vs without GWI symptoms (OR = 1.21, 95% CI = 1.06–1.39, *p* = 0.006), albeit with a smaller difference in magnitude (8.83 vs. 8.21 pg/mL, *p* = 0.63) than prior studies. Despite inconsistencies, across studies IL-1α and TNF-RI remain cytokines of interest with respect to GWI symptoms. Our data also suggest that some cytokines may be more indicative of deployment status and not GWI, specifically IL-1β.

**Table 4:** Review of serum/plasma cytokines in Gulf War Illness (GWI).

Author, year	Number of subjects	Deployment status	Plasma versus Serum	Elevated in GWI	Decreased in GWI	No differences
Alshelh, 2020	n = 48, 15 GWI; 33 controls	Cases all deployed, controls included deployed veterans and civilians	Plasma			✓
Janulewicz, 2019	n = 13, 8 GWI; 5 controls	Only deployed veterans included in study	Plasma	TNF-RI		
Cheng, 2020	n = 49, 33 GWI; 16 controls	Only deployed veterans included in study	Plasma	TNF-RII		
Khaiboullina, 2015	n = 79, 37 GWI; 42 controls	Cases all deployed; controls were civilians	Serum	IFN-γ, IL-5, IL-17A, IL-17F, IL-33, FGF, CCL11	TNF-α, IL-4, IL-7, IL-13, IL-25, CCL5, CXCL8	
Johnson, 2016	n = 85, 57 GWI; 28 controls	Only deployed veterans included in study	Plasma	CRP, leptin, BDNF, MMP-9	H-FABP, MMP-2	

*GWI* Gulf War Illness; *TNF* Tumor Necrosis Factor; *IFN* Interferon; *IL* Interleukin; *FGF* Fibroblast Growth Factor; *CCL* Chemokine (C–C motif) ligand; *CXCL* Chemokine (C-X-C motif) ligand; *CRP* C-reactive protein, *BDNF* Brain-derived neurotrophic factor, *MMP* Matrix metalloproteinase; *H-FABP* Heart-type fatty binding protein.

It is important to consider, however, than many factors, including the higher frequency of a PTSD diagnosis and anti-depressant use in individuals with GWI symptoms vs controls, may have impacted our cytokine data. Overall, inconsistencies have been noted regarding cytokine levels in PTSD, with some studies reporting higher levels of IL-1β, IL-6, and TNF-α, and others no significant differences in these cytokines, between cases and controls^38^. Cytokines levels have also been noted to change with anti-depressant use, with decreases in IFN-γ and TNF-α and an increase in IL-10 reported in animal models of depression^39^. By contrast, in our study, IL-1β levels were lower in individuals with GWI symptoms, who had higher rates of PTSD.

As with all studies, our findings must be interpreted considering the study limitations which included a geographically defined study population tested at one time point. As such, we cannot comment on whether our OCT measures change with time. A strength of the study is examining both deployment status and GWI symptoms when considering our findings. Despite the limitations, our study suggests differences in RNFL (temporal thickening) and GCL‒IPL (inferior temporal thinning) in individuals with versus without GWI symptoms. While this data needs to be replicated in diverse populations, these data set the foundation of using non-invasive OCT imaging as a diagnostic biomarker for GWI and perhaps as a tool for monitoring disease progression and effect of therapy.

## Supplementary Information

Supplementary Information 1.

Supplementary Information 2.

Supplementary Information 3.

## Data Availability

The datasets analyzed during the current study are not publicly available due to participant privacy but may be found in the attached supplementary files. Any additional data will be available from the corresponding author on reasonable request.

## References

[1] Baksh BS, Zayan KL, Goldhardt R (2021). Ocular manifestations and biomarkers of Gulf War Illness in US veterans. Sci. Rep..

[2] Sanchez V, Baksh BS, Cabrera K (2021). Dry eye symptoms and signs in US veterans with Gulf War illness. Am. J. Ophthalmol..

[3] Krengel MH, Zundel CG, Heeren T (2022). Health symptom trajectories and neurotoxicant exposures in Gulf War veterans: The Ft devens cohort. Environ. Health.

[4] Lei K, Kunnel A, Metzger-Smith V (2020). Diminished corticomotor excitability in Gulf War Illness related chronic pain symptoms; Evidence from TMS study. Sci. Rep..

[5] Haines DD, Ottenweller JE, Dickens BF (2017). Activity of paraoxonase/arylesterase and butyrylcholinesterase in peripheral blood of gulf war era veterans with neurologic symptom complexes or post-traumatic stress disorder. J. Occup. Environ. Med..

[6] Khaiboullina SF, DeMeirleir KL, Rawat S (2015). Cytokine expression provides clues to the pathophysiology of Gulf War illness and myalgic encephalomyelitis. Cytokine.

[7] Hubbard NA, Hutchison JL, Motes MA (2014). Central executive dysfunction and deferred prefrontal processing in veterans with Gulf War illness. Clin. Psychol. Sci..

[8] Zhang Y, Avery T, Vakhtin AA (2020). Brainstem atrophy in Gulf War Illness. Neurotoxicology.

[9] Addiego FM, Zajur K, Knack S (2021). Subcortical brain segment volumes in Gulf War illness and myalgic encephalomyelitis/chronic fatigue syndrome. Life Sci..

[10] Bhende M, Shetty S, Parthasarathy MK, Ramya S (2018). Optical coherence tomography: A guide to interpretation of common macular diseases. Indian J. Ophthalmol..

[11] Theotoka D, Wall S, Galor A (2022). The use of high resolution optical coherence tomography (HR-OCT) in the diagnosis of ocular surface masqueraders. Ocul. Surf..

[12] Wu CW, Chen HY, Chen JY, Lee CH (2022). Glaucoma detection using support vector machine method based on spectralis OCT. Diagnostics (Basel).

[13] Coppola G, Di Renzo A, Ziccardi L (2015). Optical coherence tomography in Alzheimer's disease: A meta-analysis. PLoS ONE.

[14] Garcia-Martin E, Satue M, Otin S (2014). Retina measurements for diagnosis of Parkinson disease. Retina.

[15] Sanchez-Dalmau B, Martinez-Lapiscina EH, Pulido-Valdeolivas I (2018). Predictors of vision impairment in multiple sclerosis. PLoS ONE.

[16] Saidha S, Sotirchos ES, Oh J (2013). Relationships between retinal axonal and neuronal measures and global central nervous system pathology in multiple sclerosis. JAMA Neurol..

[17] Steele L (2000). Prevalence and patterns of Gulf War illness in Kansas veterans: Association of symptoms with characteristics of person, place, and time of military service. Am. J. Epidemiol..

[18] Vujosevic S, Parra MM, Hartnett ME (2022). Optical coherence tomography as retinal imaging biomarker of neuroinflammation/neurodegeneration in systemic disorders in adults and children. Eye (Lond).

[19] Birkeldh U, Manouchehrinia A, Hietala MA (2017). The temporal retinal nerve fiber layer thickness is the most important optical coherence tomography estimate in multiple sclerosis. Front. Neurol..

[20] Kersten HM, Danesh-Meyer HV, Kilfoyle DH, Roxburgh RH (2015). Optical coherence tomography findings in Huntington's disease: A potential biomarker of disease progression. J. Neurol..

[21] La Morgia C, Barboni P, Rizzo G (2013). Loss of temporal retinal nerve fibers in Parkinson disease: A mitochondrial pattern?. Eur. J. Neurol..

[22] Wang D, Liu HL, Du YY (2021). Characterisation of thickness changes in the peripapillary retinal nerve fibre layer in patients with Leber's hereditary optic neuropathy. Br. J. Ophthalmol..

[23] Petzold A, Balcer LJ, Calabresi PA (2017). Retinal layer segmentation in multiple sclerosis: A systematic review and meta-analysis. Lancet Neurol..

[24] Barboni P, Savini G, Feuer WJ (2012). Retinal nerve fiber layer thickness variability in Leber hereditary optic neuropathy carriers. Eur. J. Ophthalmol..

[25] Pietroboni AM, Carandini T, Dell'Arti L (2020). Evidence of retinal anterograde neurodegeneration in the very early stages of multiple sclerosis: A longitudinal OCT study. Neurol Sci..

[26] Cheung CY, Ong YT, Hilal S (2015). Retinal ganglion cell analysis using high-definition optical coherence tomography in patients with mild cognitive impairment and Alzheimer's disease. J. Alzheimers Dis..

[27] Sari ES, Koc R, Yazici A (2015). Ganglion cell-inner plexiform layer thickness in patients with Parkinson disease and association with disease severity and duration. J. Neuroophthalmol..

[28] Evangelou N, Konz D, Esiri MM (2001). Size-selective neuronal changes in the anterior optic pathways suggest a differential susceptibility to injury in multiple sclerosis. Brain.

[29] Jonas JB, Gusek GC, Naumann GO (1988). Optic disc morphometry in chronic primary open-angle glaucoma. I. Morphometric intrapapillary characteristics. Graefes Arch. Clin. Exp. Ophthalmol..

[30] Carelli V, Ross-Cisneros FN, Sadun AA (2004). Mitochondrial dysfunction as a cause of optic neuropathies. Prog. Retin Eye Res..

[31] Curcio CA, Messinger JD, Sloan KR (2011). Human chorioretinal layer thicknesses measured in macula-wide, high-resolution histologic sections. Investig. Ophthalmol. Vis. Sci..

[32] Chao LL, Rothlind JC, Cardenas VA (2010). Effects of low-level exposure to sarin and cyclosarin during the 1991 Gulf War on brain function and brain structure in US veterans. Neurotoxicology.

[33] Solomon SG (2021). Retinal ganglion cells and the magnocellular, parvocellular, and koniocellular subcortical visual pathways from the eye to the brain. Handb. Clin. Neurol..

[34] Wu X, Shetty AK, Reddy DS (2021). Long-term changes in neuroimaging markers, cognitive function and psychiatric symptoms in an experimental model of Gulf War illness. Life Sci..

[35] Alshelh Z, Albrecht DS, Bergan C (2020). In-vivo imaging of neuroinflammation in veterans with Gulf War illness. Brain Behav. Immun..

[36] Janulewicz PA, Seth RK, Carlson JM (2019). The Gut-microbiome in Gulf War veterans: A preliminary report. Int. J. Environ. Res .Public Health.

[37] Broderick G, Kreitz A, Fuite J (2011). A pilot study of immune network remodeling under challenge in Gulf War Illness. Brain Behav. Immun..

[38] Zhang L, Hu XZ, Li X (2020). Potential chemokine biomarkers associated with PTSD onset, risk and resilience as well as stress responses in US military service members. Transl. Psychiatry.

[39] Kenis G, Maes M (2002). Effects of antidepressants on the production of cytokines. Int. J. Neuropsychopharmacol..

