# Supplementary material for: Ocular and inflammatory markers associated with Gulf War illness symptoms
Source: Sci Rep. 2023 Mar 2;13:3512. doi: 10.1038/s41598-023-30544-9 (PMC9981620; doi:10.1038/s41598-023-30544-9)
Supplement: Supplementary file 1 — Supplementary Information 1. [file 41598_2023_30544_MOESM1_ESM.pdf]

Supplementary Figure 1: Kansas Questionnaire Survey for Gulf War Illness Symptoms

Kansas criteria for Gulf War Illness

Instructions: Symptoms (1) ***must have started during or after the Gulf War*** and (2) must have been present within the last year. Please score your symptoms in the past 6 months as either none, mild, moderate, or severe. **Only rate symptoms that began during or after the Gulf War.**

**Symptoms after the Gulf War**	**Severity of symptom in past 6 months**			
**Fatigue / Sleep problems**				
Feeling unwell after exercise or exertion	None	Mild	Moderate	Severe
Fatigue	None	Mild	Moderate	Severe
Moderate or multiple fatigue symptoms	None	Mild	Moderate	Severe
Problems staying asleep or falling asleep	None	Mild	Moderate	Severe
Not feeling rested after sleep	None	Mild	Moderate	Severe
**Pain symptoms**				
Pain in muscles	None	Mild	Moderate	Severe
Body pain. Hurts all over	None	Mild	Moderate	Severe
Moderate or multiple pain symptoms	None	Mild	Moderate	Severe
Pain in joints	None	Mild	Moderate	Severe
**Neurologic / Cognitive / Mood symptoms**				
Night sweats	None	Mild	Moderate	Severe
Feeling irritable or angry outbursts	None	Mild	Moderate	Severe
Problems remembering recent information	None	Mild	Moderate	Severe
Symptomatic response to chemicals, odors	None	Mild	Moderate	Severe
Difficulty concentrating	None	Mild	Moderate	Severe
Trouble finding words when speaking	None	Mild	Moderate	Severe
Moderate or multiple neurological symptoms	None	Mild	Moderate	Severe

Low tolerance for heat or cold	None	Mild	Moderate	Severe
Feeling, dizzy, lightheaded, or faint	None	Mild	Moderate	Severe
Feeling down or depressed	None	Mild	Moderate	Severe
Headaches	None	Mild	Moderate	Severe
Eyes very sensitive to light	None	Mild	Moderate	Severe
Blurred or double vision	None	Mild	Moderate	Severe
Numbness or tingling in hands or feet	None	Mild	Moderate	Severe
Tremors or shaking	None	Mild	Moderate	Severe
**Gastrointestinal symptoms**				
Nausea or upset stomach	None	Mild	Moderate	Severe
Abdominal pain or cramping	None	Mild	Moderate	Severe
Moderate or multiple gastrointestinal symptoms	None	Mild	Moderate	Severe
Diarrhea	None	Mild	Moderate	Severe
**Respiratory symptoms**				
Difficulty breathing or catching your breath	None	Mild	Moderate	Severe
Moderate or multiple respiratory symptoms	None	Mild	Moderate	Severe
Wheezing	None	Mild	Moderate	Severe
Persistent cough without a cold	None	Mild	Moderate	Severe
**Skin symptoms**				
Rashes	None	Mild	Moderate	Severe
Moderate or multiple skin symptoms	None	Mild	Moderate	Severe

Adapted from:

Steele L. Prevalence and patterns of Gulf War illness in Kansas veterans: association of symptoms with characteristics of person, place, and time of military service. *Am J Epidemiol.* 2000;152(10):992-1002.

PATIENT HEALTH QUESTIONNAIRE (PHQ-9)

NAME: ______________________________ DATE: ______________

Over the last *2 weeks,* how often have you been bothered by any of the following problems?
(use "✓" to indicate your answer)

	Not at all	Several days	More than half the days	Nearly every day
**1.** Little interest or pleasure in doing things	0	1	2	3
**2.** Feeling down, depressed, or hopeless	0	1	2	3
**3.** Trouble falling or staying asleep, or sleeping too much	0	1	2	3
**4.** Feeling tired or having little energy	0	1	2	3
**5.** Poor appetite or overeating	0	1	2	3
**6.** Feeling bad about yourself—or that you are a failure or have let yourself or your family down	0	1	2	3
**7.** Trouble concentrating on things, such as reading the newspaper or watching television	0	1	2	3
**8.** Moving or speaking so slowly that other people could have noticed. Or the opposite — being so figety or restless that you have been moving around a lot more than usual	0	1	2	3
**9.** Thoughts that you would be better off dead, or of hurting yourself	0	1	2	3
add columns		+	+	

(Healthcare professional: For interpretation of TOTAL, please refer to accompanying scoring card). TOTAL:

**10.** If you checked off *any problems,* how *difficult* have these problems made it for you to do your work, take care of things at home, or get along with other people?	Not difficult at all	________
	Somewhat difficult	________
	Very difficult	________
	Extremely difficult	________

Copyright © 1999 Pfizer Inc. All rights reserved. Reproduced with permission. PRIME-MD© is a trademark of Pfizer Inc.

A2663B 10-04-2005

PHQ-9 Patient Depression Questionnaire

For initial diagnosis:

1. Patient completes PHQ-9 Quick Depression Assessment.
2. If there are at least 4 ✓s in the shaded section (including Questions #1 and #2), consider a depressive disorder. Add score to determine severity.

Consider Major Depressive Disorder

- if there are at least 5 ✓s in the shaded section (one of which corresponds to Question #1 or #2)

Consider Other Depressive Disorder

- if there are 2-4 ✓s in the shaded section (one of which corresponds to Question #1 or #2)

Note: Since the questionnaire relies on patient self-report, all responses should be verified by the clinician, and a definitive diagnosis is made on clinical grounds taking into account how well the patient understood the questionnaire, as well as other relevant information from the patient.
Diagnoses of Major Depressive Disorder or Other Depressive Disorder also require impairment of social, occupational, or other important areas of functioning (Question #10) and ruling out normal bereavement, a history of a Manic Episode (Bipolar Disorder), and a physical disorder, medication, or other drug as the biological cause of the depressive symptoms.

To monitor severity over time for newly diagnosed patients or patients in current treatment for depression:

1. Patients may complete questionnaires at baseline and at regular intervals (eg, every 2 weeks) at home and bring them in at their next appointment for scoring or they may complete the questionnaire during each scheduled appointment.
2. Add up ✓s by column. For every ✓: Several days = 1 More than half the days = 2 Nearly every day = 3
3. Add together column scores to get a TOTAL score.
4. Refer to the accompanying **PHQ-9 Scoring Box** to interpret the TOTAL score.
5. Results may be included in patient files to assist you in setting up a treatment goal, determining degree of response, as well as guiding treatment intervention.

Scoring: add up all checked boxes on PHQ-9

For every ✓ Not at all = 0; Several days = 1;
More than half the days = 2; Nearly every day = 3

Interpretation of Total Score

Total Score	Depression Severity
1-4	Minimal depression
5-9	Mild depression
10-14	Moderate depression
15-19	Moderately severe depression
20-27	Severe depression

PHQ9 Copyright © Pfizer Inc. All rights reserved. Reproduced with permission. PRIME-MD ® is a trademark of Pfizer Inc.

A2662B 10-04-2005

ID# ____________________ Date_____________________

Appendix A

DePaul Symptom Questionnaire

Please answer the following questions.

1. What is your height?_______________________

2. What is your weight?_______________________

3. What is your date of birth?_________________________

4. What is your gender?_______________________

5. To which of the following race(s) do you belong?

☐Black, African-American

☐White

☐American Indian or Alaska Native

☐Asian or Pacific Islander

☐Other race (*Please specify*)____

6. Are you of Latino or Hispanic origin?

☐Yes ☐No

7. What is your current marital status?

☐Married or living with partner

☐Separated

☐Widowed

☐Divorced

☐Never married

8. Do you have any children?

☐Yes ☐No (*Skip to Question 9)*

8a. How many children do you have?_____________

8b. How many of your children are under 18 years old?________________________________

9. How many people live in your home?____

10. What grade or degree have you completed in school?

- ☐ Less than high school
- ☐ Some high school
- ☐ High school degree or GED
- ☐ Partial college (at least one year) or specialized training
- ☐ Standard college degree
- ☐ Graduate professional degree including masters and doctorate

11. What is your current work status? **(Check all that apply)**

- ☐ On disability
- ☐ Student
- ☐ Homemaker
- ☐ Retired
- ☐ Unemployed
- ☐ Working part-time
- ☐ Working full-time

11a. If you are on disability, for what condition do you receive disability compensation?

Please Specify____

12. What is your current occupation?

Current____

12a. If you are currently not working, what was your most recent occupation?

Most Recent_______________________________________

For the following questions (13-66), we would like to know **how often you have had each symptom** and **how much each symptom has bothered you over the last 6 months**. For each symptom please circle **one number for frequency and one number for severity**. Please fill the chart out from left to right.

**Symptoms**	*Frequency:*<br>Throughout the **past 6 months**, how **often** have you had this symptom?<br>For each symptom listed below, circle a number from:<br>**0 = none of the time**<br>**1 = a little of the time**<br>**2 = about half the time**<br>**3 = most of the time**<br>**4 = all of the time**					*Severity:*<br>Throughout the **past 6 months**, **how much** has this symptom bothered you?<br>For each symptom listed below, circle a number from:<br>**0 = symptom not present**<br>**1 = mild**<br>**2 = moderate**<br>**3 = severe**<br>**4 = very severe**				
**13) Fatigue/extreme tiredness**	0	1	2	3	4	0	1	2	3	4
**14) Dead, heavy feeling after starting to exercise**	0	1	2	3	4	0	1	2	3	4
**15) Next day soreness or fatigue after non-strenuous, everyday activities**	0	1	2	3	4	0	1	2	3	4
**16) Mentally tired after the slightest effort**	0	1	2	3	4	0	1	2	3	4
**17) Minimum exercise makes you physically tired**	0	1	2	3	4	0	1	2	3	4
**18) Physically drained or sick after mild activity**	0	1	2	3	4	0	1	2	3	4
**19) Feeling unrefreshed after you wake up in the morning**	0	1	2	3	4	0	1	2	3	4
**20) Need to nap daily**	0	1	2	3	4	0	1	2	3	4
**21) Problems falling asleep**	0	1	2	3	4	0	1	2	3	4
**22) Problems staying asleep**	0	1	2	3	4	0	1	2	3	4
**23) Waking up early in the morning (e.g. 3am)**	0	1	2	3	4	0	1	2	3	4
**24) Sleep all day and stay awake all night**	0	1	2	3	4	0	1	2	3	4
**25) Pain or aching in your muscles**	0	1	2	3	4	0	1	2	3	4
**26) Pain/stiffness/tenderness in more than one joint without swelling or redness**	0	1	2	3	4	0	1	2	3	4
**27) Eye pain**	0	1	2	3	4	0	1	2	3	4

**Symptoms**	*Frequency:* Throughout the **past 6 months**, **how often** have you had this symptom? For each symptom listed below, circle a number from: **0 = none of the time** **1 = a little of the time** **2 = about half the time** **3 = most of the time** **4 = all of the time**					*Severity:* Throughout the **past 6 months**, **how much** has this symptom bothered you? For each symptom listed below, circle a number from: **0 = symptom not present** **1 = mild** **2 = moderate** **3= severe** **4 = very severe**				
**28) Chest pain**	0	1	2	3	4	0	1	2	3	4
**29) Bloating**	0	1	2	3	4	0	1	2	3	4
**30) Abdomen/stomach pain**	0	1	2	3	4	0	1	2	3	4
**31) Headaches**	0	1	2	3	4	0	1	2	3	4
**32) Muscle twitches**	0	1	2	3	4	0	1	2	3	4
**33) Muscle weakness**	0	1	2	3	4	0	1	2	3	4
**34) Sensitivity to noise**	0	1	2	3	4	0	1	2	3	4
**35) Sensitivity to bright lights**	0	1	2	3	4	0	1	2	3	4
**36) Problems remembering things**	0	1	2	3	4	0	1	2	3	4
**37) Difficulty paying attention for a long period of time**	0	1	2	3	4	0	1	2	3	4
**38) Difficulty finding the right word to say or expressing thoughts**	0	1	2	3	4	0	1	2	3	4
**39) Difficulty understanding things**	0	1	2	3	4	0	1	2	3	4
**40) Only able to focus on one thing at a time**	0	1	2	3	4	0	1	2	3	4
**41) Unable to focus vision and/or attention**	0	1	2	3	4	0	1	2	3	4
**42) Loss of depth perception**	0	1	2	3	4	0	1	2	3	4
**43) Slowness of thought**	0	1	2	3	4	0	1	2	3	4
**44) Absent-mindedness or forgetfulness**	0	1	2	3	4	0	1	2	3	4
**45) Bladder problems**	0	1	2	3	4	0	1	2	3	4
**46) Irritable bowel problems**	0	1	2	3	4	0	1	2	3	4

Symptoms	*Frequency:*<br>Throughout the **past 6 months**, **how often** have you had this symptom?<br>For each symptom listed below, circle a number from:<br>**0 = none of the time**<br>**1 = a little of the time**<br>**2 = about half the time**<br>**3 = most of the time**<br>**4 = all of the time**					*Severity:*<br>Throughout the **past 6 months**, **how much** has this symptom bothered you?<br>For each symptom listed below, circle a number from:<br>**0 = symptom not present**<br>**1 = mild**<br>**2 = moderate**<br>**3= severe**<br>**4 = very severe**				
**47) Nausea**	0	1	2	3	4	0	1	2	3	4
**48) Feeling unsteady on your feet, like you might fall**	0	1	2	3	4	0	1	2	3	4
**49) Shortness of breath or trouble catching your breath**	0	1	2	3	4	0	1	2	3	4
**50) Dizziness or fainting**	0	1	2	3	4	0	1	2	3	4
**51) Irregular heart beats**	0	1	2	3	4	0	1	2	3	4
**52) Losing or gaining weight without trying**	0	1	2	3	4	0	1	2	3	4
**53) No appetite**	0	1	2	3	4	0	1	2	3	4
**54) Sweating hands**	0	1	2	3	4	0	1	2	3	4
**55) Night sweats**	0	1	2	3	4	0	1	2	3	4
**56) Cold limbs (e.g. arms, legs, hands)**	0	1	2	3	4	0	1	2	3	4
**57) Feeling chills or shivers**	0	1	2	3	4	0	1	2	3	4
**58) Feeling hot or cold for no reason**	0	1	2	3	4	0	1	2	3	4
**59) Feeling like you have a high temperature**	0	1	2	3	4	0	1	2	3	4
**60) Feeling like you have a low temperature**	0	1	2	3	4	0	1	2	3	4
**61) Alcohol intolerance**	0	1	2	3	4	0	1	2	3	4
**62) Sore throat**	0	1	2	3	4	0	1	2	3	4
**63) Tender/sore lymph nodes**	0	1	2	3	4	0	1	2	3	4
**64) Fever**	0	1	2	3	4	0	1	2	3	4
**65) Flu-like symptoms**	0	1	2	3	4	0	1	2	3	4
**66) Some smells, foods, medications, or chemicals make you feel sick**	0	1	2	3	4	0	1	2	3	4

67. Have you **always** had persistent or recurring **fatigue/energy problems**, even back to your earliest memories as a child? (By persistent or recurring, we mean that the fatigue/energy problems are usually ongoing and constant, but sometimes there are good periods and bad periods.)

☐Yes ☐No ☐Not having a problem with fatigue/energy

68. Since your **fatigue/energy related illness** began, do your headaches either happen more often, feel worse or more severe, or are they in a different place or spot?

☐Yes ☐No ☐Not having a problem with fatigue/energy

69. How long ago did your problem with **fatigue/energy** begin?

☐Less than 6 months

☐6-12 months

☐1-2 years

☐Longer than 2 years

☐Had problem with fatigue/energy since childhood or adolescence

☐Not having a problem with fatigue/energy

70. Have you been diagnosed with Chronic Fatigue Syndrome or Myalgic Encephalomyelitis?

☐Yes ☐No

70a. If yes, what year were you diagnosed?__________________

70b. Do you currently have a diagnosis of Chronic Fatigue Syndrome or Myalgic Encephalomyelitis?

☐Yes ☐No

70c. Who diagnosed you with Chronic Fatigue Syndrome or Myalgic Encephalomyelitis?

☐Medical Doctor ☐Alternative Practitioner ☐Self-Diagnosed

70d. Have any of your family members been diagnosed with Chronic Fatigue Syndrome or Myalgic Encephalomyelitis?

☐Yes ☐No

If yes, please list their relation to you and current age_____________________________

____

71. Did you experience any of the following symptoms regularly and repeatedly in the months and years <u>before</u> your fatigue/energy problems began?

☐Sore throat

☐Tender/sore lymph nodes

☐Unrefreshing sleep

☐Impaired memory and concentration

☐Prolonged fatigue following physical or mental exertion

☐Muscle pain

☐Headaches

☐Joint Pain

☐Not having a problem with fatigue/energy

72. If you rest, does your problem with **fatigue/energy** go away? **(Check one)**

☐ Entirely

☐ Partially

☐ My fatigue/energy problem is not improved by rest *(Skip to Question 73)*

☐I am not having a problem with fatigue/energy *(Skip to Question 73)*

72a. How long do you have to rest for your problem with **fatigue/energy** to entirely or partially go away?

☐less than 30 minutes ☐30 to 59 minutes ☐1-2 hours ☐more than 2 hours

73. If you were to become exhausted after actively participating in extracurricular activities, sports, or outings with friends, would you recover within an hour or two after the activity ended?

☐Yes ☐No

74. Do you reduce your activity level to avoid experiencing problems with **fatigue/energy**?

☐Yes ☐No ☐Not having a problem with fatigue/energy

75. Do you experience a worsening of your **fatigue/energy related illness** after engaging in minimal physical effort?

☐Yes ☐No ☐Not having a problem with fatigue/energy

75a. Do you experience a worsening of your **fatigue/energy related illness** after engaging in mental effort?

☐Yes ☐No

75b. If you feel worse after activities, how long does this last? **(Check one)**

☐ 1 hour or less ☐ 2-3 Hrs ☐ 4-10 Hrs ☐ 11-13 Hrs

☐14-23 Hrs ☐ More than 24 Hrs (Please specify ________)

76. Are you currently engaging in any form of exercise?

☐Yes *(Skip to Question 77)* ☐ No

76a. If you do not exercise, why aren't you exercising? **(Check all boxes that you agree with)**

☐ Not interested

☐ No time

☐ Would like to but cannot because of problems with fatigue/energy

☐ Cannot because exercise makes symptoms worse

77. Over what period of time did your **fatigue/energy related illness,** develop? **(Check one)**

- ☐ Within 24 hours
- ☐ Over 1 week
- ☐ Over 1 month
- ☐ Over 2-6 months
- ☐ Over 7-12 months
- ☐ Over 1-2 years
- ☐ Longer than 2 years
- ☐ Had problem with fatigue/energy since childhood or adolescence
- ☐ I am not ill

78. How would you describe the course of your **fatigue/energy related illness? (Check one)**

- ☐ Constantly getting worse
- ☐ Constantly improving
- ☐ Persisting (no change)
- ☐ Relapsing & remitting (having “good” periods with no symptoms & “bad” periods)
- ☐ Fluctuating (symptoms periodically get better and get worse, but never disappear completely)
- ☐ No Symptoms/I am not ill

79. Which statement best describes your **fatigue/energy related illness** during the **<u>last 6 months</u>**? **(Check one)**

- ☐ I am not able to work or do anything, and I am bedridden.
- ☐ I can walk around the house, but I cannot do light housework.
- ☐ I can do light housework, but I cannot work part-time.
- ☐ I can only work part-time at work or on some family responsibilities.
- ☐ I can work full time, but I have no energy left for anything else.
- ☐ I can work full time and finish some family responsibilities but I have no energy left for anything else.

☐I can do all work or family responsibilities without any problems with my energy.

80. Did your **fatigue/energy related illness** start after you experienced any of the following? **(Check one or more and please specify)**

☐An infectious illness________________________________

☐An accident________________________________

☐A trip or vacation________________________________

☐An immunization (shot at doctor's office)________________________________

☐Surgery________________________________

☐Severe stress (bad or unhappy event(s))________________________________

☐Other________________________________

☐I am not ill

81. Have you ever consulted a medical doctor or health professional about your **fatigue/energy** problem?

☐Yes ☐No *(Skip to Question 83)*

82. Do you currently have a medical doctor overseeing your **fatigue/energy** problem?

☐Yes ☐No

83. Do you have any medical illness (es) that might be causing your symptoms?

☐Yes ☐No *(Skip to Question 84)*

83a. What medical illnesses do you have?

Illness name(s) and year it began:________________________________

____

____

____

83b. For which of these conditions are you currently receiving treatment?________________

____

____

____

84. Are you currently taking any medications (over the counter or prescription)?

☐ Yes ☐ No*(Skip to Question 86)*

84a. What medications are you taking? ____

____

85. Do you think any medication(s) is (are) causing your problem with **fatigue/energy**?

☐Yes ☐No *(Skip to Question 86)*

☐I do not have a problem with fatigue/energy *(Skip to Question 86)*

85a. Please specify which medications:____

____

86. Have you ever been diagnosed and/or treated for any of the following: **(Check all that apply and write year (s) experienced, years treated, and medication (if applicable) in the blank)**

☐Major depression____

☐Major depression with melancholic or psychotic features__________________________

☐Bipolar disorder (Manic-depression)____

☐Anxiety____

☐Schizophrenia____

☐Eating disorder____

☐Substance abuse____

☐Multiple chemical sensitivities____

☐Fibromyalgia____

☐Allergies____

☐Other (*Please specify*)____

☐No diagnosis/treatment

87. What do you think is the cause of your problem with **fatigue/energy**? **(Check one)**

☐Definitely physical

☐Mainly physical

☐Equally physical and psychological

☐Mainly psychological

☐Definitely psychological

☐No problem with fatigue/energy

88. Do you think anything specific in your personal life or environment accounts for your problem with **fatigue/energy**?

☐Yes ☐No *(Skip to Question 89)*

☐I do not have a problem with fatigue/energy (*Skip to Question 89)*

88a. Please specify:____

89. In the **past 4 weeks**, approximately how many hours per week have you spent doing:

Household related activities?____________hours per week

Social/Recreational related activities?_____hours per week

Family related activities?_______________hours per week

Work related activities?________________hours per week

90. In the **past 4 weeks**, have you had to reduce the number of hours you previously spent (prior to your illness) on occupational, social or family activities because of your health or problems with **fatigue/energy**?

☐ Yes ☐ No*(Skip to Question 91)* ☐Not having a problem with fatigue/energy

90a. **Before your fatigue/energy related illness**, approximately how many hours did you used to spend on:

Household related activities? ____________hours per week

Social/Recreational related activities? _____hours per week

Family related activities? _______________hours per week

Work related activities?_________________hours per week

NOTE: For those people who are NOT having a problem with fatigue/energy, please answer questions 91-96 assuming that a score of 100= having abundant energy that allows one to work full-time and perform daily chores.

91. Please rate the amount of **energy** you had available **yesterday,** using a scale from 1 to 100 where 1 = no energy and 100 = your pre-illness energy level_______________

92. Please rate the amount of **energy** you expended (used) **yesterday,** using a scale from 1 to 100 where 1 = no energy and 100 = your pre-illness energy expended______________

93. Please rate the amount of **fatigue** you had **yesterday,** using a scale from 1 to 100 where 1 = no fatigue and 100 = severe fatigue______________

94. For the **past week,** please rate the amount of **energy** you had available using a scale from 1 to 100 where 1=no energy and 100=your pre-illness energy level________________

95. For the **past week,** please rate the amount of **energy** you have expended (used) using a scale from 1 to 100 where 1 = no energy and 100 = your pre-illness energy expended____________

96. For the **past week,** please rate the amount of **fatigue** you have had using a scale from 1 to 100 where 1 = no fatigue and 100 = severe fatigue _________________

MOS SURVEY

INSTRUCTIONS:

This survey asks for your views about your health. This information will help keep track of how you feel and how well you are able to do your usual activities. Answer every question by marking the answer as indicated. If you are unsure about how to answer a question, please give the best answer you can.

1. In general, would you say your health is: ***(Please circle one)***

   Excellent.. 1
   Very good... 2
   Good 3
   Fair 4
   Poor.. 5

2. **<u>Compared to one year ago,</u>** how would you rate your health in general now? ***(Please circle one)***

   Much better than one year ago 1
   Somewhat better now than one year ago 2
   About the same as one year ago 3
   Somewhat worse now than one year ago................. 4
   Much worse now than one year ago 5

3. The following items are about activities you might do during a typical day. Does your health now limit you in these activities? If so, how much?

**Activities**	**Yes, Limited A Lot**	**Yes, Limited A Little**	**No, Not Limited At All**
**Vigorous activities**: running, lifting heavy objects, participating in strenuous sports	1	2	3
**Moderate activities**: moving a table, pushing a vacuum cleaner, bowling, playing golf	1	2	3
Lifting or carrying groceries	1	2	3
Climbing **several** flights of stairs	1	2	3
Climbing **one** flight of stairs	1	2	3
Bending, kneeling, or stooping	1	2	3
Walking **more than a mile**	1	2	3
Walking **several blocks**	1	2	3
Walking **one** block	1	2	3
Bathing or dressing yourself	1	2	3

4. During the **past 4 weeks**, have you had any of the following problems with your work or other regular daily activities as a result of your **physical health**?

**Problems**	**Yes**	**No**
Cut down on the **amount of time** you spent on work or other activities	1	2
**Accomplished less** than you would like	1	2
Were limited in the **kind** of work or other activities	1	2
Had **difficulty** performing the work or other activities (For example, it took extra effort)	1	2

5. During the **past 4 weeks**, have you had any of the following problems with your work or other regular daily activities **as a result of any emotional problems** (such as feeling depressed or anxious)?

**Problems**	**Yes**	**No**
Cut down the **amount of time** you spent on work or other activities	1	2
**Accomplished less** than you would like	1	2
Didn't do work or other activities as **carefully** as usual	1	2

6. During the **past 4 weeks**, to what extent has your physical health or emotional problems interfered with your normal social activities with family, neighbors, or groups? ***(Please circle one)***

Not at all 1
Slightly 2
Moderately 3
Quite a bit 4
Extremely 5

7. How much bodily pain have you had during the **past 4 weeks**?

None 1
Very mild 2
Mild 3
Moderate 4
Severe 5
Very Severe 6

8. During the **past 4 weeks**, how much did pain interfere with your normal work (including both work outside the home and housework)?

Not at all 1
Slightly 2
Moderately 3
Quite a bit 4
Extremely 5

9. These questions are about how you feel and how things have been with you **during the past 4 weeks**. For each question, please give the one answer that comes closest to the way you have been feeling. How much of the time **during the past 4 weeks**-

**Questions**	**All of the Time**	**Most of the Time**	**A Good Bit of the Time**	**Some of the Time**	**A Little of the Time**	**None of the Time**
Did you feel full of pep?	1	2	3	4	5	6
Have you been a nervous person?	1	2	3	4	5	6
Have you felt so down in the dumps that nothing could cheer you up?	1	2	3	4	5	6
Have you felt calm and peaceful?	1	2	3	4	5	6
Did you have a lot of energy?	1	2	3	4	5	6
Have you felt down-hearted and blue?	1	2	3	4	5	6
Did you feel worn out?	1	2	3	4	5	6
Have you been a happy person?	1	2	3	4	5	6
Did you feel tired?	1	2	3	4	5	6

10. During the **past 4 weeks**, how much of the time has your physical health or emotional problems interfered with your social activities (like visiting with friends, relatives, etc.)?

All of the time 1
Most of the time.. 2
Some of the time 3
A little of the time.. 4
None of the time 5

11. How **TRUE** or **FALSE** is each of following statements for you?

**Statements**	**Definitely True**	**Mostly True**	**Don't Know**	**Mostly False**	**Definitely False**
I seem to get sick a little easier than other people	1	2	3	4	5
I am as healthy as anybody I know	1	2	3	4	5
I expect my health to get worse	1	2	3	4	5
My health is excellent	1	2	3	4	5

New Clinical Fibromyalgia Diagnostic Criteria – Part 1.

To answer the following questions, patients should take into consideration

- how you felt the **past week**,
- while taking your current therapies and treatments, and
- exclude your pain or symptoms from other known illnesses such as arthritis, Lupus, Sjogren's, etc.

Determining Your Widespread Pain Index (WPI)
The WPI Index score from Part 1 is between 0 and 19.

Check each area you have felt pain in over the past week.

- ☐ Shoulder girdle, left
- ☐ Shoulder girdle, right
- ☐ Upper arm, left
- ☐ Upper arm, right
- ☐ Lower arm, left
- ☐ Lower arm, right
- ☐ Hip (buttock) left
- ☐ Hip (buttock) right
- ☐ Upper leg left
- ☐ Upper leg right
- ☐ Lower leg left
- ☐ Lower leg right
- ☐ Jaw left
- ☐ Jaw right
- ☐ Chest
- ☐ Abdomen
- ☐ Neck
- ☐ Upper back
- ☐ Lower back
- ☐ None of these areas

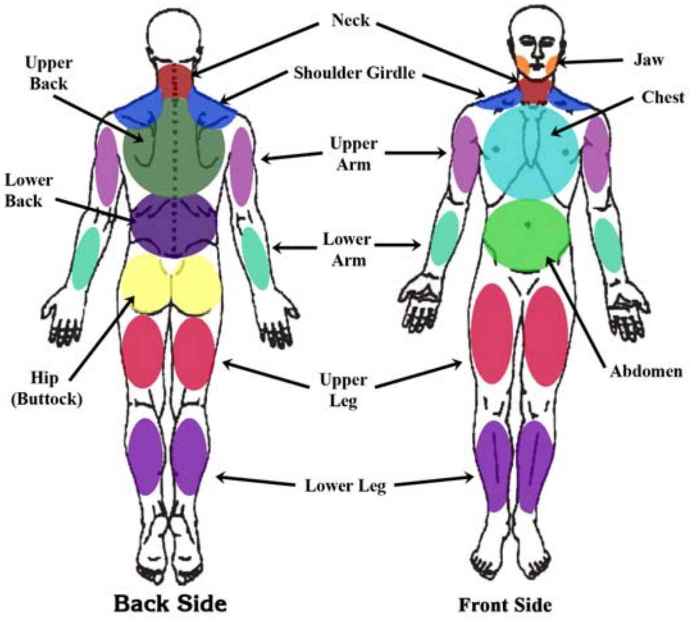

Count up the number of areas checked and enter your Widespread Pain Index or WPI score score here ______.

Symptom Severity Score (SS score) - Part 2a.

Indicate your level of symptom severity over the past week using the following scale.

Fatigue

- ☐ 0 = No problem
- ☐ 1 = Slight or mild problems; generally mild or intermittent
- ☐ 2 = Moderate; considerable problems; often present and/or at a moderate level
- ☐ 3 = Severe: pervasive, continuous, life disturbing problems

Waking unrefreshed

- ☐ 0 = No problem
- ☐ 1 = Slight or mild problems; generally mild or intermittent
- ☐ 2 = Moderate; considerable problems; often present and/or at a moderate level
- ☐ 3 = Severe: pervasive, continuous, life disturbing problems

Cognitive symptoms

- ☐ 0 = No problem
- ☐ 1 = Slight or mild problems; generally mild or intermittent
- ☐ 2 = Moderate; considerable problems; often present and/or at a moderate level
- ☐ 3 = Severe: pervasive, continuous, life disturbing problems

Tally your score for Part 2a (not the number of checkmarks) and enter it here ______.

Symptom Severity Score (SS score)- Part 2b

Check each of the following OTHER SYMPTOMS that you have experienced over the past week?

☐ Muscle pain	☐ Nervousness	☐ Loss/change in taste
☐ Irritable bowel syndrome	☐ Chest pain	☐ Seizures
☐ Fatigue/tiredness	☐ Blurred vision	☐ Dry eyes
☐ Thinking or remembering problem	☐ Fever	☐ Shortness of breath
☐ Muscle Weakness	☐ Diarrhea	☐ Loss of appetite
☐ Headache	☐ Dry mouth	☐ Rash
☐ Pain/cramps in abdomen	☐ Itching	☐ Sun sensitivity
☐ Numbness/tingling	☐ Wheezing	☐ Hearing difficulties
☐ Dizziness	☐ Raynauld's	☐ Easy bruising
☐ Insomnia	☐ Hives/welts	☐ Hair loss
☐ Depression	☐ Ringing in ears	☐ Frequent urination
☐ Constipation	☐ Vomiting	☐ Painful urination
☐ Pain in upper abdomen	☐ Heartburn	☐ Bladder spasms
☐ Nausea	☐ Oral ulcers	

Count up the number of symptoms checked above.
*If you tallied:

0 symptoms	Give yourself a score of 0
1 to 10	Give yourself a score of 1
11 to 24	Give yourself a score of 2
25 or more	Give yourself a score of 3

Enter your score for Part 2b here _____.

Now add Part 2a AND 2b scores, and enter ____.
This is your Symptom Severity Score (SS score), which can range from 0 to 12.

What Your Scores Mean

A patient meets the diagnostic criteria for fibromyalgia if the following 3 conditions are met:

1a. The WPI score (Part 1) is greater than or equal to 7 **AND** the SS score (Part 2a & b) is greater than or equal to 5

OR

1b. The WPI score (Part 1) is from 3 to 6 **AND** the SS score (Part 2a & b) is greater than or equal to 9.

2. Symptoms have been present at a similar level for at least 3 months.

3. You do not have a disorder that would otherwise explain the pain.

For example:
If your WPI (Part 1) was 9 and your SS score (Parts 2a & b) was 6, then you **would meet** the new FM diagnostic criteria.

If your WPI (Part 1) was 5 and your SS score (Parts 2a & b) was 7, then you **would NOT** meet the new FM diagnostic criteria.

*The new FM diagnostic criteria did not specify the number of "Other Symptoms" required to score the point rankings from 0 to 3. Therefore, we estimated the number of symptoms needed to meet the authors' descriptive categories of:

0 = No symptoms
1 = Few symptoms
2 = A moderate number
3 = A great deal of symptoms

* Wolfe F, *et al*. Arthritis Care Res 62(5):600-610, 2010.

For information about Fibromyalgia Network, call our office Monday through Friday, 9:00 a.m. to 5:00 p.m. (PST) at (800) 853-2929 or visit us online at www.fmnetnews.com.

This survey is not meant to substitute for a diagnosis by a medical professional. Patients should not diagnose themselves. Patients should always consult their medical professional for advice and treatment. This survey is intended to give you insight into research on the diagnostic criteria and measurement of symptom severity for fibromyalgia.

Modified Fatigue Impact Scale (MFIS)

Fatigue is a feeling of physical tiredness and lack of energy that many people experience from time to time. But people who have medical conditions like MS experience stronger feelings of fatigue more often and with greater impact than others.

Following is a list of statements that describe the effects of fatigue. Please read each statement carefully, the circle the one number that best indicates how often fatigue has affected you in this way during the past 4 weeks. (If you need help in marking your responses, tell the interviewer the number of the best response.) Please answer every question. If you are not sure which answer to select choose the one answer that comes closest to describing you. Ask the interviewer to explain any words or phrases that you do not understand.

Because of my fatigue during the past 4 weeks

		Never	Rarely	Sometimes	Often	Almost Always
1.	I have been less alert.	0	1	2	3	4
2.	I have had difficulty paying attention for long periods of time.	0	1	2	3	4
3.	I have been unable to think clearly.	0	1	2	3	4
4.	I have been clumsy and uncoordinated.	0	1	2	3	4
5.	I have been forgetful.	0	1	2	3	4
6.	I have had to pace myself in my physical activities.	0	1	2	3	4
7.	I have been less motivated to do anything that requires physical effort.	0	1	2	3	4
8.	I have been less motivated to participate in social activities.	0	1	2	3	4
9.	I have been limited in my ability to do things away from home.	0	1	2	3	4
10.	I have trouble maintaining physical effort for long periods.	0	1	2	3	4
11.	I have had difficulty making decisions.	0	1	2	3	4
12.	I have been less motivated to do anything that requires thinking	0	1	2	3	4
13.	My muscles have felt weak	0	1	2	3	4
14.	I have been physically uncomfortable.	0	1	2	3	4
15.	I have had trouble finishing tasks that require thinking.	0	1	2	3	4
16.	I have had difficulty organizing my thoughts when doing things at home or at work.	0	1	2	3	4
17.	I have been less able to complete tasks that require physical effort.	0	1	2	3	4

		Never	Rarely	Sometimes	Often	Almost Always
18.	My thinking has been slowed down.	0	1	2	3	4
19.	I have had trouble concentrating.	0	1	2	3	4
20.	I have limited my physical activities.	0	1	2	3	4
21.	I have needed to rest more often or for longer periods.	0	1	2	3	4

Instructions for Scoring the MFIS

Items on the MFIS can be aggregated into three subscales (physical, cognitive, and psychosocial), as well as into a total MFIS score. All items are scaled so that higher scores indicate a greater impact of fatigue on a person's activities.

Physical Subscale

This scale can range from 0 to 36. It is computed by adding raw scores on the following items: 4+6+7+10+13+14+17+20+21. 0

Cognitive Subscale

This scale can range from 0 to 40. It is computed by adding raw scores on the following items: 1+2+3+5+11+12+15+16+18+19. 0

Psychosocial Subscale

This scale can range from 0 to 8. It is computed by adding raw scores on the following items: 8+9. 0

Total MFIS Score

The total MFIS score can range from 0 to 84. It is computed by adding scores on the physical, cognitive, and psychosocial subscales. 0

SF-12® Patient Questionnaire

Patient Initials _____ _____ ______ **Date of Birth:** ___/___/___ **Patkey:** ______

Surgeon Name: ____ **Date:** ____________

Examination Period: _____ Preop (1) _____ Immediate Postop (2) _____ 1 Year (3) _____ 3 Year (4) _____ 5 Year (5) _____ Other (specify) (6): _____________

SF-12®:
This information will help your doctors keep track of how you feel and how well you are able to do your usual activities. Answer every question by placing a check mark on the line in front of the appropriate answer. It is not specific for arthritis. If you are unsure about how to answer a question, please give the best answer you can and make a written comment beside your answer.

1. In general, would you say your health is:
   _____ Excellent (1)
   _____ Very Good (2)
   _____ Good (3)
   _____ Fair (4)
   _____ Poor (5)

The following two questions are about activities you might do during a typical day. Does YOUR HEALTH NOW LIMIT YOU in these activities? If so, how much?

2. MODERATE ACTIVITIES, such as moving a table, pushing a vacuum cleaner, bowling, or playing golf:
   _____ Yes, Limited A Lot (1)
   _____ Yes, Limited A Little (2)
   _____ No, Not Limited At All (3)

3. Climbing SEVERAL flights of stairs:
   _____ Yes, Limited A Lot (1)
   _____ Yes, Limited A Little (2)
   _____ No, Not Limited At All (3)

During the PAST 4 WEEKS have you had any of the following problems with your work or other regular activities AS A RESULT OF YOUR PHYSICAL HEALTH?

4. ACCOMPLISHED LESS than you would like:
   _____ Yes (1)
   _____ No (2)

5. Were limited in the KIND of work or other activities:
   _____ Yes (1)
   _____ No (2)

Surgeon Initials __________ **Date:** _____________

Patient Initials _____ _____ ______ **Date of Birth:** ___/___/___ **Patkey:** ______

Surgeon Name: ____ **Date:** ___________

Examination Period: _____ Preop (1) _____ 3 Year (4)
_____ Immediate Postop (2) _____ 5 Year (5)
_____ 1 Year (3) _____ Other (specify) (6): ___________

SF-12® Cont'd:

During the PAST 4 WEEKS, were you limited in the kind of work you do or other regular activities AS A RESULT OF ANY EMOTIONAL PROBLEMS (such as feeling depressed or anxious)?

6. ACCOMPLISHED LESS than you would like:
   _____ Yes (1)
   _____ No (2)

7. Didn't do work or other activities as CAREFULLY as usual:
   _____ Yes (1)
   _____ No (2)

8. During the PAST 4 WEEKS, how much did PAIN interfere with your normal work (including both work outside the home and housework)?
   _____ Not At All (1)
   _____ A Little Bit (2)
   _____ Moderately (3)
   _____ Quite A Bit (4)
   _____ Extremely (5)

The next three questions are about how you feel and how things have been DURING THE PAST 4 WEEKS. For each question, please give the one answer that comes closest to the way you have been feeling. How much of the time during the PAST 4 WEEKS –

9. Have you felt calm and peaceful?
   _____ All of the Time (1)
   _____ Most of the Time (2)
   _____ A Good Bit of the Time (3)
   _____ Some of the Time (4)
   _____ A Little of the Time (5)
   _____ None of the Time (6)

Surgeon Initials __________ **Date:** _____________

Patient Initials _____ _____ ______ **Date of Birth:** ___/___/___ **Patkey:** ______

Surgeon Name: ____ **Date:** ____________

Examination Period:
_____ Preop (1)
_____ Immediate Postop (2)
_____ 1 Year (3)
_____ 3 Year (4)
_____ 5 Year (5)
_____ Other (specify) (6): ____________

SF-12® Cont'd:

10. Did you have a lot of energy?
_____ All of the Time (1)
_____ Most of the Time (2)
_____ A Good Bit of the Time (3)
_____ Some of the Time (4)
_____ A Little of the Time (5)
_____ None of the Time (6)

11. Have you felt downhearted and blue?
_____ All of the Time (1)
_____ Most of the Time (2)
_____ A Good Bit of the Time (3)
_____ Some of the Time (4)
_____ A Little of the Time (5)
_____ None of the Time (6)

12. During the PAST 4 WEEKS, how much of the time has your PHYSICAL HEALTH OR EMOTIONAL PROBLEMS interfered with your social activities (like visiting with friends, relatives, etc.)?
_____ All of the Time (1)
_____ Most of the Time (2)
_____ A Good Bit of the Time (3)
_____ Some of the Time (4)
_____ A Little of the Time (5)
_____ None of the Time (6)

Surgeon Signature____ **Date**________________________

SF-12® Health Survey © 1994, 2002 by Medical Outcomes Trust and QualityMetric Incorporated. All Rights Reserved
SF-12® is a registered trademark of Medical Outcomes Trust

Name:____ Date: _________________

Pittsburgh Sleep Quality Index (PSQI)

Instructions: The following questions relate to your usual sleep habits during the past month only. Your answers should indicate the most accurate reply for the majority of days and nights in the past month. **Please answer all questions.**

1. During the past month, what time have you usually gone to bed at night? ___________________
2. During the past month, how long (in minutes) has it usually taken you to fall asleep each night? __________
3. During the past month, what time have you usually gotten up in the morning? ___________________
4. During the past month, how many hours of actual sleep did you get at night? (This may be different than the number of hours you spent in bed.) ___________________

5. During the past month, how often have you had trouble sleeping because you…	Not during the past month	Less than once a week	Once or twice a week	Three or more times a week
a. Cannot get to sleep within 30 minutes				
b. Wake up in the middle of the night or early morning				
c. Have to get up to use the bathroom				
d. Cannot breathe comfortably				
e. Cough or snore loudly				
f. Feel too cold				
g. Feel too hot				
h. Have bad dreams				
i. Have pain				
j. Other reason(s), please describe:				
6. During the past month, how often have you taken medicine to help you sleep (prescribed or "over the counter")?				
7. During the past month, how often have you had trouble staying awake while driving, eating meals, or engaging in social activity?				
	No problem at all	Only a very slight problem	Somewhat of a problem	A very big problem
8. During the past month, how much of a problem has it been for you to keep up enough enthusiasm to get things done?				
	Very good	Fairly good	Fairly bad	Very bad
9. During the past month, how would you rate your sleep quality overall?				

	No bed partner or room mate	Partner/room mate in other room	Partner in same room but not same bed	Partner in same bed
10. Do you have a bed partner or room mate?				
	Not during the past month	Less than once a week	Once or twice a week	Three or more times a week
If you have a room mate or bed partner, ask him/her how often in the past month you have had:				
a. Loud snoring				
b. Long pauses between breaths while asleep				
c. Legs twitching or jerking while you sleep				
d. Episodes of disorientation or confusion during sleep				
e. Other restlessness while you sleep, please describe:				

Scoring the PSQI

The order of the PSQI items has been modified from the original order in order to fit the first 9 items (which are the only items that contribute to the total score) on a single page. Item 10, which is the second page of the scale, does not contribute to the PSQI score.

In scoring the PSQI, seven component scores are derived, each scored 0 (no difficulty) to 3 (severe difficulty). The component scores are summed to produce a global score (range 0 to 21). Higher scores indicate worse sleep quality.

Component 1: Subjective sleep quality—question 9

Response to Q9	Component 1 score
Very good	0
Fairly good	1
Fairly bad	2
Very bad	3

Component 1 score:_____

Component 2: Sleep latency—questions 2 and 5a

Response to Q2	Component 2/Q2 subscore
≤ 15 minutes	0
16-30 minutes	1
31-60 minutes	2
> 60 minutes	3

Response to Q5a	Component 2/Q5a subscore
Not during past month	0
Less than once a week	1
Once or twice a week	2
Three or more times a week	3

Sum of Q2 and Q5a subscores	Component 2 score
0	0
1-2	1
3-4	2
5-6	3

Component 2 score:_____

Component 3: Sleep duration—question 4

Response to Q4	Component 3 score
> 7 hours	0
6-7 hours	1
5-6 hours	2
< 5 hours	3

Component 3 score:_____

Component 4: Sleep efficiency—questions 1, 3, and 4

Sleep efficiency = (# hours slept/# hours in bed) X 100%

hours slept—question 4

hours in bed—calculated from responses to questions 1 and 3

Sleep efficiency	Component 4 score
> 85%	0
75-84%	1
65-74%	2
< 65%	3

Component 4 score:_____

Component 5: Sleep disturbance—questions 5b-5j

Questions 5b to 5j should be scored as follows:	
Not during past month	0
Less than once a week	1
Once or twice a week	2
Three or more times a week	3

Sum of 5b to 5j scores	Component 5 score
0	0
1-9	1
10-18	2
19-27	3

Component 5 score:_____

Component 6: Use of sleep medication—question 6

Response to Q6	Component 6 score
Not during past month	0
Less than once a week	1
Once or twice a week	2
Three or more times a week	3

Component 6 score:_____

Component 7: Daytime dysfunction—questions 7 and 8

Response to Q7	Component 7/Q7 subscore
Not during past month	0
Less than once a week	1
Once or twice a week	2
Three or more times a week	3

Response to Q8	Component 7/Q8 subscore
No problem at all	0
Only a very slight problem	1
Somewhat of a problem	2
A very big problem	3

Sum of Q7 and Q8 subscores	Component 7 score
0	0
1-2	1
3-4	2
5-6	3

Component 7 score:_____

Global PSQI Score: Sum of seven component scores:___________

Copyright notice: The Pittsburgh Sleep Quality Index (PSQI) is copyrighted by Daniel J. Buysse, M.D. Permission has been granted to reproduce the scale on this website for clinicians to use in their practice and for researchers to use in non-industry studies. For other uses of the scale, the owner of the copyright should be contacted.

Citation: Buysse, DJ, Reynolds CF, Monk TH, Berman SR, Kupfer DJ: The Pittsburgh Sleep Quality Index (PSQI): A new instrument for psychiatric research and practice. Psychiatry Research 28:193-213, 1989

Based on the scale below, please rate the intensity of eye pain at this moment (scale 0 to 10, 10 being the worst).

Left Eye:	0	1	2	3	4	5	6	7	8	9	10
Right Eye:	0	1	2	3	4	5	6	7	8	9	10

Based on the scale below, please rate the intensity of eye pain on average over the last week (scale 0 to 10, 10 being the worst).

Left Eye:	0	1	2	3	4	5	6	7	8	9	10
Right Eye:	0	1	2	3	4	5	6	7	8	9	10

Based on the scale below, please rate the intensity of eye pain at its worst over the last week (scale 0 to 10, 10 being the worst).

Left Eye:	0	1	2	3	4	5	6	7	8	9	10
Right Eye:	0	1	2	3	4	5	6	7	8	9	10

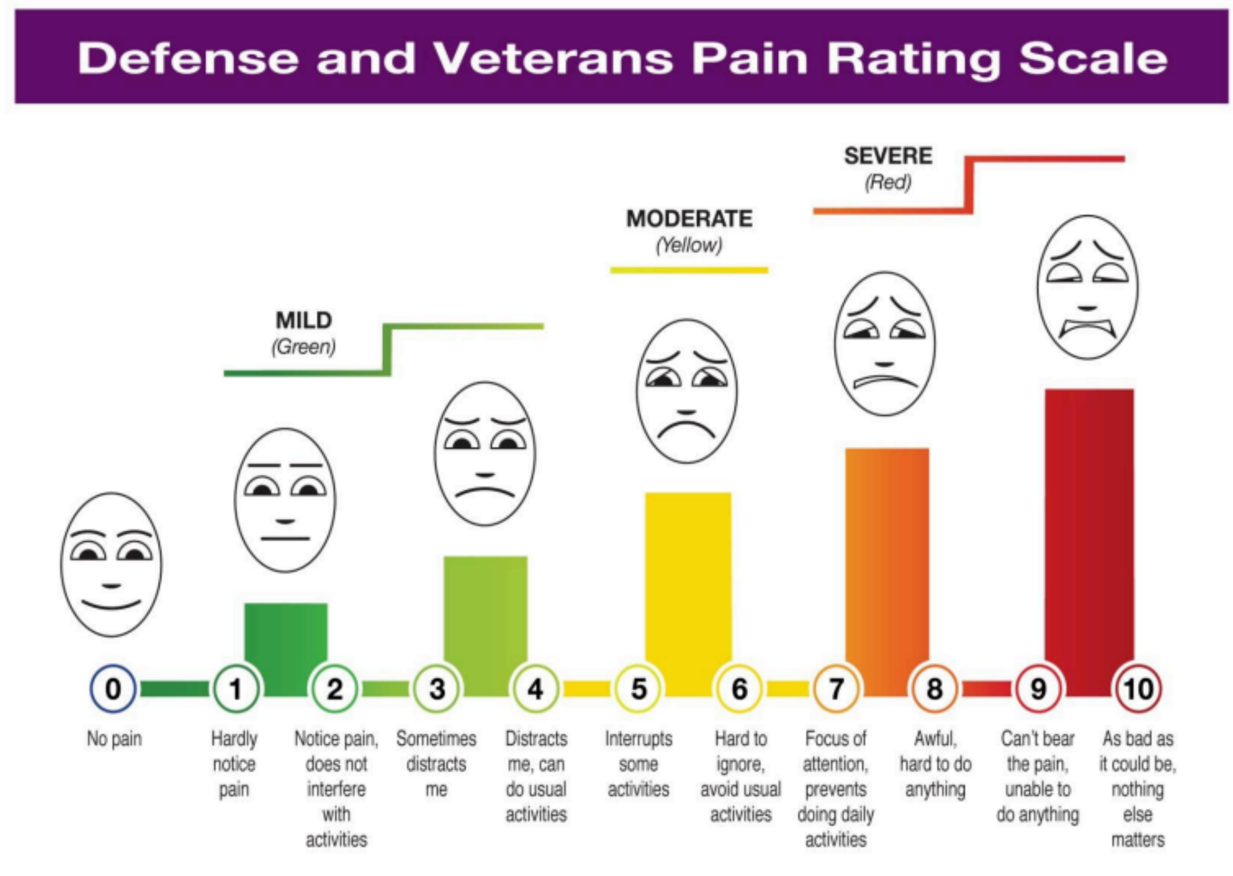

DEQ 5

1. Questions about **EYE DISCOMFORT**:
   a. During a typical day in the past month, **how often** did your eyes feel discomfort?

      0 Never
      1 Rarely
      2 Sometimes
      3 Frequently
      4 Constantly

   b. When your eyes felt discomfort, **how intense was this feeling of discomfort** at the end of the day, within two hours of going to bed?

Never Have It	Not at all Intense				Very Intense
0	1	2	3	4	5

2. Questions about **EYE DRYNESS**
   a. During a typical day in the past month, **how often** did your eyes feel dry?

      0 Never
      1 Rarely
      2 Sometimes
      3 Frequently
      4 Constantly

   b. When your eyes felt dry, **how intense was this feeling of dryness** at the end of the day, within two hours of going to bed?

Never Have It	Not at all Intense				Very Intense
0	1	2	3	4	5

3. Question about **WATERY EYES**:
   During a typical day in the past month, **how often** did your eyes look or feel excessively watery?

   0 Never
   1 Rarely
   2 Sometimes
   3 Frequently
   4 Constantly

Neuropathic Pain Symptom Inventory - Eye

We wish to know if you feel spontaneous eye pain, that is, pain without any stimulation. For each of the following questions, please select the number that best describes your *average spontaneous pain severity during the past 24 h*. Select the number 0 if you have not felt such pain (circle one number only).

Q1. Does your eye pain feel like burning?

No burning	0	1	2	3	4	5	6	7	8	9	10	Worst burning imaginable

Q2. Does your eye pain feel like squeezing?

No squeezing	0	1	2	3	4	5	6	7	8	9	10	Worst squeezing imaginable

Q3. Does your eye pain feel like pressure?

No pressure	0	1	2	3	4	5	6	7	8	9	10	Worst pressure imaginable

Q4. *During the past 24 h*, your spontaneous pain has been present:
Select *the response* that best describes your case

Permanently ____
Between 8 and 12 hours ____
Between 4 and 7 hours ____
Between 1 and 3 hours ____
Less than 1 hour ____

We wish to know if you have brief attacks of eye pain. For each of the following questions, please select the number that best describes the *average severity of your painful attacks during the past 24 h*. Select the number 0 if you have not felt such pain (circle one number only).

Q5. Does your eye pain feel like electric shocks?

No electric shocks	0	1	2	3	4	5	6	7	8	9	10	Worst electric shocks imaginable

Q6. Does your eye pain feel like stabbing?

No stabbing	0	1	2	3	4	5	6	7	8	9	10	Worst stabbing imaginable

Q7. *During the past 24 h*, how many of these pain attacks have you had?
Select *the response* that best describes your case

More than 20 ____
Between 11 and 20 ____
Between 6 and 10 ____
Between 1 and 5 ____

No pain attack ____

We wish to know if you feel eye pain provoked or increased by wind, light, or contact with cold/hot. For each of the following questions, please select the number that best describes the *average severity of your provoked pain during the past 24 h*. Select the number 0 if you have not felt such pain (circle one number only).

Q8. Is your eye pain provoked or increased by wind?

No pain	0	1	2	3	4	5	6	7	8	9	10	Worst pain imaginable

Q9. Is your eye pain provoked or increased by light?

No pain	0	1	2	3	4	5	6	7	8	9	10	Worst pain imaginable

Q10. Is your eye pain provoked or increased by *contact* with something cold or hot (air conditioned/warm weather)?

No pain	0	1	2	3	4	5	6	7	8	9	10	Worst pain imaginable

We wish to know if you feel abnormal eye sensations. For each of the following questions, please select the number that best describes the *average severity of your abnormal sensations during the past 24 h*. Select the number 0 if your have not felt such sensation (circle one number only).

Q11. Do you feel pins and needles?

No pins and needles	0	1	2	3	4	5	6	7	8	9	10	Worst pins and needles imaginable

Q12. Do you feel tingling?

No tingling	0	1	2	3	4	5	6	7	8	9	10	Worst tingling imaginable

RESULTS

	Total intensity score	Subscores	
1.	Q1=	Burning (superficial) spontaneous pain: Q1=	/10
2.	(Q2+Q3)=	Pressing (deep) spontaneous pain: (Q2+Q3)/2=	/10
3.	(Q5+Q6)=	Paroxysmal pain: (Q5+Q6)/2=	/10
4.	(Q8+Q9+Q10)=	Evoked pain: (Q8+Q9+Q10)/3=	/10
5.	(Q11+Q12)=	Paresthesia/dysesthesia: (Q11+Q12)/2=	/10
(1+2+3+4+5)= /100			

Ocular Surface Disease Index© (OSDI©)[2]

Ask your patient the following 12 questions, and circle the number in the box that best represents each answer. Then, fill in boxes A, B, C, D, and E according to the instructions beside each.

HAVE YOU EXPERIENCED ANY OF THE FOLLOWING *DURING THE LAST WEEK*:

	All of the time	Most of the time	Half of the time	Some of the time	None of the time
1. Eyes that are sensitive to light?	4	3	2	1	0
2. Eyes that feel gritty?	4	3	2	1	0
3. Painful or sore eyes?	4	3	2	1	0
4. Blurred vision?	4	3	2	1	0
5. Poor vision?	4	3	2	1	0

Subtotal score for answers 1 to 5 (A)

HAVE PROBLEMS WITH YOUR EYES LIMITED YOU IN PERFORMING ANY OF THE FOLLOWING *DURING THE LAST WEEK*:

	All of the time	Most of the time	Half of the time	Some of the time	None of the time	
6. Reading?	4	3	2	1	0	N/A
7. Driving at night?	4	3	2	1	0	N/A
8. Working with a computer or bank machine (ATM)?	4	3	2	1	0	N/A
9. Watching TV?	4	3	2	1	0	N/A

Subtotal score for answers 6 to 9 (B)

HAVE YOUR EYES FELT UNCOMFORTABLE IN ANY OF THE FOLLOWING SITUATIONS *DURING THE LAST WEEK*:

	All of the time	Most of the time	Half of the time	Some of the time	None of the time	
10. Windy conditions?	4	3	2	1	0	N/A
11. Places or areas with low humidity (very dry)?	4	3	2	1	0	N/A
12. Areas that are air conditioned?	4	3	2	1	0	N/A

Subtotal score for answers 10 to 12 (C)

ADD SUBTOTALS A, B, AND C TO OBTAIN D (D = SUM OF SCORES FOR ALL QUESTIONS ANSWERED) (D)

TOTAL NUMBER OF QUESTIONS ANSWERED (DO NOT INCLUDE QUESTIONS ANSWERED N/A) (E)

Please turn over the questionnaire to calculate the patient's final OSDI© score.

Schiffman RM, Christianson MD, Jacobsen G, Hirsch JD, Reis BL. Reliability and validity of the Ocular Surface Disease Index. Arch Ophthalmol. 2000;118:615-621

Convergence Insufficiency Symptom Survey (CISS)

Name:________________________________ Date:___ /___ /____

Clinician/Assistant instructions: Pose the following questions exactly as written. If the patient responds with "yes" - please qualify with frequency choices. Do not give examples.

Patient instructions: Please answer the following questions about how your eyes feel when reading or doing close work.

Possible Subjective Symptoms	Frequency: Never (0)	Infrequently/ not very often (1)	Sometimes (2)	Fairly often (3)	Always (4)
1. Do your eyes feel tired when reading or doing close work?					
2. Do your eyes feel uncomfortable when reading or doing close work?					
3. Do you have headaches when reading or doing close work?					
4. Do you feel sleepy when reading or doing close work?					
5. Do you lose concentration when reading or doing close work?					
6. Do you have trouble remembering what you have read?					
7. Do you have double vision when reading or doing close work?					
8. Do you see the words move, jump, swim or appear to float on the page when reading or doing close work?					
9. Do you feel like you read slowly?					
10. Do your eyes ever hurt when reading or doing close work?					
11. Do your eyes ever feel sore when reading or doing close work?					
12. Do you feel a "pulling" feeling around your eyes when reading or doing close work?					
13. Do you notice the words blurring or coming in and out of focus when reading or doing close work?					
14. Do you lose your place while reading or doing close work?					
**Total score** ________	__x 0	__ x 1	__x 2	___ x 3	__x 4

For Children (< age 21) total score = **16 or higher** is suggestive of convergence insufficiency.
For Adults total score = **21 or higher** is suggestive of convergence insufficiency.

Reference: Borsting EJ, Rouse MW, Mitchell GL, et al and the CITT group. Validity and reliability of the revised convergence insufficiency symptom survey in children. Optometry and Vision Science 2003; 80(12):832-838.

American Optometric Association

Penlight Red/Green (PLRG) Procedure For Screening of Convergence Insufficiency

Convergence Insufficiency is a condition in which a patient finds it difficult to maintain alignment of the eyes on a near object. Inability to sustain convergence may cause a person to look with just one eye at a time, or to see double.

The PLRG procedure is a near point of convergence test with good sensitivity that is relatively easy to perform in a short period of time. With the room illumination dimmed, the patient is asked to put on the red/green glasses. If the patient typically wears glasses for near, the red/green glasses are placed over the patient's eyewear.

The penlight is presented directly in front of the patient at a distance of 24 inches. The penlight is held along the midline and slightly below eye level. Ask the patient "How Many Lights Do You See?" If the patient has normal convergence to that distance, the expected response is "one." Tell the patient that you are going to slowly move the penlight toward their nose and ask them to report if they ever see two lights instead of one. At some point, while you are slowly moving the penlight inward, the patient should report seeing two colored lights, one red and one green. This is the convergence break point. The penlight is then moved slowly away from the nose until the patient reports seeing one light again.

Repeat this procedure three times and record the convergence break point for the third measurement only. The reason why you repeat the procedure three times is because the Near Point of Convergence tends to recede (move outward) over time due to fatigue, particularly when the patient has convergence insufficiency.

5-Step Screening for Convergence Insufficiency

Place red/green glasses on patient

▼

Instruct patient to watch light and state when they see a green light and a red light

▼

On the third trial, record the distance where the patient reports two lights

▼ Patient reports two lights **closer in than 4 inches** (10 cm)

▼ Patient passes screening

▼ Patient reports two lights **further out than 4 inches** (10 cm)

▼ Have patient complete Convergence Insufficiency Symptom Checklist (other side)

▼ Doctor will review data with patient

Reference: Scheiman M, Gallaway M, Frantz KA, et al. Near point of convergence: test procedure, target selection, normative data. Optometry and Vision Science 2003; 80(3):214-225.
