# Supplementary material for: Ocular and inflammatory markers associated with Gulf War illness symptoms
Source: Sci Rep. 2023 Mar 2;13:3512. doi: 10.1038/s41598-023-30544-9 (PMC9981620; doi:10.1038/s41598-023-30544-9)
Supplement: Supplementary file 2 — Supplementary Information 2. [file 41598_2023_30544_MOESM2_ESM.docx]

**Supplementary Table 1:** Average Thickness of Nerve Fiber Layer and Ganglion Cell Layer-Inner Plexiform Layer measurements between individuals with Gulf War Illness symptoms and those without Gulf War Illness symptoms.

|  | Presence of GWI symptoms | Number of Individuals | Mean Thickness (µM) | Standard Deviation  (µM) | Standard Error Mean (µM) |
| --- | --- | --- | --- | --- | --- |
| Central Macula Minimum Value | No GWI symptoms | 65 | 255.34 | 26.36 | 3.27 |
|  | GWI symptoms | 36 | 258.61 | 17.96 | 2.99 |
| Inner Superior Macular Maximum Value | No GWI symptoms | 65 | 325.03 | 16.78 | 2.08 |
|  | GWI symptoms | 36 | 325.89 | 20.42 | 3.40 |
| Outer Superior Macula Maximum Value | No GWI symptoms | 65 | 280.94 | 14.48 | 1.80 |
|  | GWI symptoms | 36 | 278.58 | 17.58 | 2.93 |
| Inner Nasal Macula Maximum Value | No GWI symptoms | 65 | 326.98 | 17.98 | 2.23 |
|  | GWI symptoms | 36 | 326.94 | 19.52 | 3.25 |
| Outer Nasal Macula Maximum Value | No GWI symptoms | 65 | 296.71 | 15.55 | 1.93 |
|  | GWI symptoms | 36 | 294.47 | 19.33 | 3.22 |
| Inner Inferior Macula Maximum Value | No GWI symptoms | 65 | 321.71 | 16.12 | 2.00 |
|  | GWI symptoms | 36 | 320.00 | 18.95 | 3.16 |
| Outer Inferior Macula Maximum Value | No GWI symptoms | 65 | 270.11 | 15.91 | 1.97 |
|  | GWI symptoms | 36 | 267.61 | 16.07 | 2.68 |
| Inner Temporal Macula Minimum Value | No GWI symptoms | 65 | 307.94 | 16.60 | 2.06 |
|  | GWI symptoms | 36 | 306.25 | 13.92 | 2.32 |
| Outer Temporal Macula Minimum Value | No GWI symptoms | 65 | 259.09 | 16.56 | 2.05 |
|  | GWI symptoms | 36 | 259.06 | 11.99 | 2.00 |
| Superior NFL Minimum Value | No GWI symptoms | 65 | 109.58 | 13.28 | 1.65 |
|  | GWI symptoms | 36 | 110.86 | 14.93 | 2.49 |
| Nasal NFL Minimum Value | No GWI symptoms | 65 | 69.31 | 11.20 | 1.39 |
|  | GWI symptoms | 36 | 66.81 | 10.79 | 1.80 |
| Inferior NFL Minimum Value | No GWI symptoms | 65 | 115.98 | 15.25 | 1.89 |
|  | GWI symptoms | 36 | 119.75 | 19.53 | 3.26 |
| Temporal NFL Maximum Value | No GWI symptoms | 64 | 61.14 | 10.02 | 1.25 |
|  | GWI symptoms | 36 | 68.44 | 22.76 | 3.79 |
| Temporal NFL Minimum Value | No GWI symptoms | 64 | 55.80 | 10.23 | 1.28 |
|  | GWI symptoms | 36 | 61.33 | 11.52 | 1.92 |
| Superior GCL-IPL Minimum Value | No GWI symptoms | 63 | 77.87 | 11.08 | 1.40 |
|  | GWI symptoms | 36 | 78.00 | 11.94 | 1.99 |
| Superior Nasal GCL-IPL Minimum Value | No GWI symptoms | 63 | 77.89 | 13.01 | 1.64 |
|  | GWI symptoms | 36 | 75.89 | 17.43 | 2.90 |
| Inferior Nasal GCL-IPL Minimum Value | No GWI symptoms | 63 | 78.05 | 9.67 | 1.22 |
|  | GWI symptoms | 36 | 76.06 | 13.28 | 2.21 |
| Superior Nasal GCL-IPL Minimum Value | No GWI symptoms | 63 | 82.67 | 7.49 | 0.94 |
|  | GWI symptoms | 36 | 83.42 | 9.05 | 1.51 |
| Inferior GCL-IPL Maximum Value | No GWI symptoms | 63 | 80.03 | 7.15 | 0.90 |
|  | GWI symptoms | 36 | 78.39 | 9.77 | 1.63 |
| Inferior Temporal GCL-IPL Maximum Value | No GWI symptoms | 63 | 82.11 | 6.75 | 0.85 |
|  | GWI symptoms | 36 | 81.64 | 7.68 | 1.28 |
| Inferior Temporal GCL-IPL Minimum Value | No GWI symptoms | 63 | 79.21 | 7.22 | 0.91 |
|  | GWI symptoms | 36 | 77.53 | 10.58 | 1.76 |
| Superior Temporal GCL-IPL Minimum Value | No GWI symptoms | 63 | 77.46 | 7.95 | 1.00 |
|  | GWI symptoms | 36 | 76.78 | 10.59 | 1.77 |
| Superior Temporal GCL-IPL Maximum Value | No GWI symptoms | 63 | 81.14 | 6.96 | 0.88 |
|  | GWI symptoms | 36 | 81.56 | 6.86 | 1.14 |
| Central Macula Maximum Value | No GWI symptoms | 65 | 262.78 | 28.11 | 3.49 |
|  | GWI symptoms | 36 | 269.97 | 31.04 | 5.17 |
| Inner Superior Macula Minimum Value | No GWI symptoms | 65 | 319.52 | 17.22 | 2.14 |
|  | GWI symptoms | 36 | 318.56 | 17.42 | 2.90 |
| Outer Superior Macula Maximum Value | No GWI symptoms | 65 | 274.94 | 13.89 | 1.72 |
|  | GWI symptoms | 36 | 272.86 | 15.31 | 2.55 |
| Inner Nasal Macula Minimum Value | No GWI symptoms | 65 | 321.08 | 18.25 | 2.26 |
|  | GWI symptoms | 36 | 320.69 | 16.66 | 2.78 |
| Outer Nasal Macula Minimum Value | No GWI symptoms | 65 | 290.25 | 16.99 | 2.11 |
|  | GWI symptoms | 36 | 288.47 | 18.28 | 3.05 |
| Inner Inferior Macula Minimum Value | No GWI symptoms | 65 | 315.95 | 16.82 | 2.09 |
|  | GWI symptoms | 36 | 313.47 | 15.45 | 2.57 |
| Outer Inferior Macula Minimum Value | No GWI symptoms | 65 | 263.68 | 13.26 | 1.64 |
|  | GWI symptoms | 36 | 262.64 | 13.48 | 2.25 |
| Inner Temporal Macula Maximum Value | No GWI symptoms | 65 | 314.15 | 16.32 | 2.02 |
|  | GWI symptoms | 36 | 314.14 | 17.24 | 2.87 |
| Outer Temporal Macula Maximum Value | No GWI symptoms | 65 | 267.08 | 15.68 | 1.95 |
|  | GWI symptoms | 36 | 265.06 | 16.85 | 2.81 |
| Superior NFL Maximum Value | No GWI symptoms | 65 | 119.82 | 14.77 | 1.83 |
|  | GWI symptoms | 36 | 120.83 | 13.38 | 2.23 |
| Nasal NFL Maximum Value | No GWI symptoms | 65 | 76.15 | 11.60 | 1.44 |
|  | GWI symptoms | 36 | 72.39 | 11.04 | 1.84 |
| Inferior NFL Maximum Value | No GWI symptoms | 65 | 124.08 | 15.17 | 1.88 |
|  | GWI symptoms | 36 | 128.19 | 22.14 | 3.69 |
| Superior GCL-IPL Maximum Value | No GWI symptoms | 63 | 82.37 | 7.14 | 0.90 |
|  | GWI symptoms | 36 | 82.89 | 8.39 | 1.40 |
| Inferior GCL-IPL Maximum Value | No GWI symptoms | 63 | 81.60 | 7.56 | 0.95 |
|  | GWI symptoms | 36 | 80.06 | 9.30 | 1.54 |
| Inferior GCL-IPL Minimum Value | No GWI symptoms | 63 | 76.89 | 8.58 | 1.08 |
|  | GWI symptoms | 36 | 74.50 | 11.93 | 1.99 |
| ^a^NFL = Nerve Fiber Layer; GCL-IPL = Ganglion Cell Layer-Inner Plexiform Layer; GWI = Gulf War Illness | | | | | |
