# Supplementary material for: Ocular and inflammatory markers associated with Gulf War illness symptoms
Source: Sci Rep. 2023 Mar 2;13:3512. doi: 10.1038/s41598-023-30544-9 (PMC9981620; doi:10.1038/s41598-023-30544-9)
Supplement: Supplementary file 3 — Supplementary Information 3. [file 41598_2023_30544_MOESM3_ESM.docx]

**Supplemental Table 2:** Mean Inflammatory Marker Concentration Levels in those with Gulf War Illness symptoms vs those without Gulf War Illness symptoms

|  | Presence of GWI symptoms | Number of Individuals | Mean Concentration  (µM) | Standard Deviation  (µM) | Standard Error Mean (µM) |
| --- | --- | --- | --- | --- | --- |
| TNF RI | No GWI symptoms | 69 | 481.27 | 173.19 | 20.85 |
|  | GWI symptoms | 36 | 418.06 | 157.56 | 26.26 |
| IL 1α | No GWI symptoms | 69 | 8.56 | 5.74 | 0.69 |
|  | GWI symptoms | 36 | 8.60 | 6.08 | 1.01 |
| IL1β | No GWI symptoms | 69 | 13.59 | 5.84 | 0.70 |
|  | GWI symptoms | 36 | 11.14 | 4.13 | 0.69 |
| IL2 | No GWI symptoms | 69 | 10.94 | 4.36 | 0.53 |
|  | GWI symptoms | 36 | 10.94 | 4.83 | 0.81 |
| IL4 | No GWI symptoms | 69 | 3.63 | 1.60 | 0.19 |
|  | GWI symptoms | 36 | 3.56 | 1.64 | 0.27 |
| IL5 | No GWI symptoms | 69 | 3.78 | 2.20 | 0.27 |
|  | GWI symptoms | 36 | 4.13 | 2.07 | 0.34 |
| IL6 | No GWI symptoms | 69 | 4.42 | 2.55 | 0.31 |
|  | GWI symptoms | 36 | 4.44 | 2.19 | 0.36 |
| IL8 | No GWI symptoms | 69 | 2.71 | 1.16 | 0.14 |
|  | GWI symptoms | 36 | 2.97 | 4.14 | 0.69 |
| IFN-g | No GWI symptoms | 69 | 9.07 | 3.72 | 0.45 |
|  | GWI symptoms | 36 | 9.18 | 2.05 | 0.34 |
| IL12 | No GWI symptoms | 69 | 3.67 | 2.81 | 0.34 |
|  | GWI symptoms | 36 | 5.63 | 7.97 | 1.33 |
| IL13 | No GWI symptoms | 69 | 2.52 | 1.18 | 0.14 |
|  | GWI symptoms | 36 | 2.69 | 0.92 | 0.15 |
| IL23 | No GWI symptoms | 69 | 138.79 | 244.53 | 29.44 |
|  | GWI symptoms | 36 | 151.11 | 216.79 | 36.13 |
| IL15 | No GWI symptoms | 69 | 4.46 | 1.07 | 0.13 |
|  | GWI symptoms | 36 | 4.41 | 1.91 | 0.32 |
| IL17 | No GWI symptoms | 67 | 66.38 | 95.21 | 11.63 |
|  | GWI symptoms | 34 | 84.45 | 116.25 | 19.94 |
| IL10 | No GWI symptoms | 69 | 64.61 | 95.93 | 11.55 |
|  | GWI symptoms | 34 | 35.56 | 60.14 | 10.31 |
| TNF α | No GWI symptoms | 69 | 7.32 | 3.88 | 0.47 |
|  | GWI symptoms | 36 | 8.11 | 4.15 | 0.69 |
| TNF  RII | No GWI symptoms | 69 | 509.30 | 120.41 | 14.50 |
|  | GWI symptoms | 36 | 505.28 | 210.30 | 35.05 |
| TNFβ | No GWI symptoms | 68 | 10.34 | 7.97 | 0.97 |
|  | GWI symptoms | 35 | 8.70 | 5.04 | 0.85 |
| ^a^IL = interleukin; IFN = interferon; TNF = tumor necrosis factor; GWI = Gulf War Illness | | | | | |
